# CTLA-4 blockade shifts the B cell repertoire toward autoimmunity

**DOI:** 10.1172/JCI189074

**Published:** 2025-09-30

**Authors:** Elif Çakan, Meng Wang, Yile Dai, Adrien Mirouse, Clarence Rachel Villanueva-Pachas, Delphine Bouis, Joshua M. Boeckers, Ruchi Gera, Sally Yraita, Leslie Clapp, Ana Luisa Perdigoto, Fabien R. Delmotte, Christopher Massad, Antonietta Bacchiocchi, Aaron M. Ring, Yuval Kluger, Harriet M. Kluger, Kevan C. Herold, Eric Meffre

**Affiliations:** 1 Department of Immunobiology, Yale University School of Medicine, New Haven, Connecticut, USA.; 2 Program in Computational Biology and Bioinformatics, Yale University, New Haven, Connecticut, USA.; 3 Division of Immunology and Rheumatology, Department of Medicine, Stanford University School of Medicine, Stanford, California, USA.; 4 Division of Endocrinology and Metabolism, Department of Internal Medicine, Yale University, New Haven, Connecticut, USA.; 5 Department of Dermatology,; 6 Department of Pathology,; 7 Yale Center for Immuno-Oncology,; 8 Yale Cancer Center, and; 9 Department of Medicine, Yale School of Medicine, New Haven, Connecticut, USA.

**Keywords:** Autoimmunity, Immunology, Cancer immunotherapy, Diabetes, Tolerance

## Abstract

Checkpoint inhibitors targeting CTLA-4 and PD-1 revolutionized the treatment of cancer patients, but their use is limited by the emergence of immune-related adverse events (irAEs). We assessed autoreactive B cell frequencies in the blood of cancer patients before and after treatment with checkpoint inhibitors by testing the reactivity of recombinant antibodies cloned from single B cells. We found that anti–PD-1 and anti–CTLA-4 combination therapy induced the emergence of autoreactive mature naive B cells, whereas central B cell tolerance remained functional. In contrast, anti–PD-1 alone did not alter autoreactive B cell counterselection. Anti–CTLA-4 injections in humanized mice also resulted in the production of autoreactive B cells, whereas anti–PD-1 did not. We conclude that CTLA-4 but not PD-1 is required for the removal of developing autoreactive mature naive B cells and that CTLA-4 blockade broadens the peripheral B cell repertoire, which likely contains clones that promote not only irAEs but also antitumor responses.

## Abstract 2

CTLA-4 blockade leads to a transient emergence of autoreactive mature naïve B cells that may target various tissues and be responsible for immune-related adverse events

## Introduction

Anti–CTLA-4 and anti–PD-1 monoclonal antibodies (mAbs) that antagonize the inhibitory function of these 2 molecules led to dramatic improvement of treatment for many types of cancers. Anti–PD-1 therapy led to more successful clinical outcome compared with anti–CTLA-4 therapy in some cancer types, including renal cancer, lung cancer, melanoma and lymphoma. For metastatic melanoma patients, anti–CTLA-4 and anti–PD-1 combination therapy is more effective than monotherapy (1). Unfortunately, the use of these remarkable checkpoint inhibitors (CPIs) is limited by the frequent emergence of immune-related adverse events (irAE), which may be life threatening. Thyroiditis and hypophysitis were identified in 40% and 13% of patients injected with anti–CTLA-4 or anti–PD-1 mAbs, respectively, but the incidence of these ailments increased to about 50% in patients treated with the combination therapy (2, 3). In addition, anti–PD-1 or anti–PD-L1 with or without anti–CTLA-4 mAbs may lead to rare development of insulin-dependent diabetes (4, 5). Despite all these observations, it remains unclear why some cancer patients develop irAE and others do not and why CPI differ in the cause of these events. Hence, additional studies are warranted to clarify the underlying tolerance mechanisms altered by CPI.

Successful B cell depletion therapies in autoimmune diseases such as anti-CD20 mAb treatment of patients with rheumatoid arthritis (RA) or multiple sclerosis (MS) and anti-CD19 CAR T cell treatment of patients with systemic lupus erythematosus (SLE) revealed the essential role for B cells in human autoimmune syndromes (6–8). These therapies eliminate vast numbers of autoreactive B cells that are produced and accumulate in these patients with autoimmune diseases (9). Indeed, while developing, autoreactive B cells in healthy individuals undergo 2 sequential steps of negative selection, first in the bone marrow and then in the periphery; these central and peripheral B cell tolerance checkpoints are impaired in patients with autoimmune diseases (9). The analysis of patients with rare genetic defects and humanized mouse models revealed that central B cell tolerance relies on the sensing of self antigens by B cell receptor (BCR) and TLR9 in immature B cells in the bone marrow, which leads to the silencing of autoreactive clones (9–11). In contrast, the peripheral B cell tolerance checkpoint, which prevents autoreactive B cells that escape central tolerance from entering the mature naive B cell compartment is regulated by T cells and, more specifically, Tregs, via cognate interactions (9, 11). We therefore investigated the impact of CTLA-4 and PD-1 blockade on the selection of developing autoreactive B cells in both cancer patients and humanized mice injected with anti–CTLA-4 and anti–PD-1 mAbs. We found that CTLA-4 but not PD-1 plays an essential role in preventing the accumulation of autoreactive mature naive B cells in the periphery, whereas neither of these CPIs alter central B cell tolerance. Hence, CTLA-4 blockade results in a transient production of autoreactive B cells, which may explain the higher incidence of irAE associated with anti–CTLA-4 alone or in combination with anti–PD-1 compared with anti–PD-1 monotherapy (2, 3).

## Results

### Anti–CTLA-4 and anti–PD-1 combination therapy specifically interferes with the peripheral removal of autoreactive B cells.

To determine if CPIs interfere with the establishment of B cell tolerance in humans, we tested the reactivity by ELISA of recombinant antibodies cloned from single CD19^+^CD27^–^CD10^+^IgM^hi^CD21^lo^ new emigrant/transitional and CD19^+^CD27^–^CD10^–^IgM^+^CD21^+^ mature naive B cells isolated from frozen PBMCs of 4 patients with cancer before and after treatment with CPI. All 4 patients had melanoma and were carefully selected for being treatment naive when checkpoint inhibitors were injected (Supplemental Table 1; supplemental material available online with this article; https://doi.org/10.1172/JCI189074DS1). The patients were also chosen for having the least additional treatment associated with the development of irAEs, such as thyroiditis and hypophysis, which developed shortly after receiving 2 rounds of combination therapy and which may interfere with our readouts (Supplemental Table 1). In addition, these patients had no previous history of autoimmune diseases.

**Figure 1:**
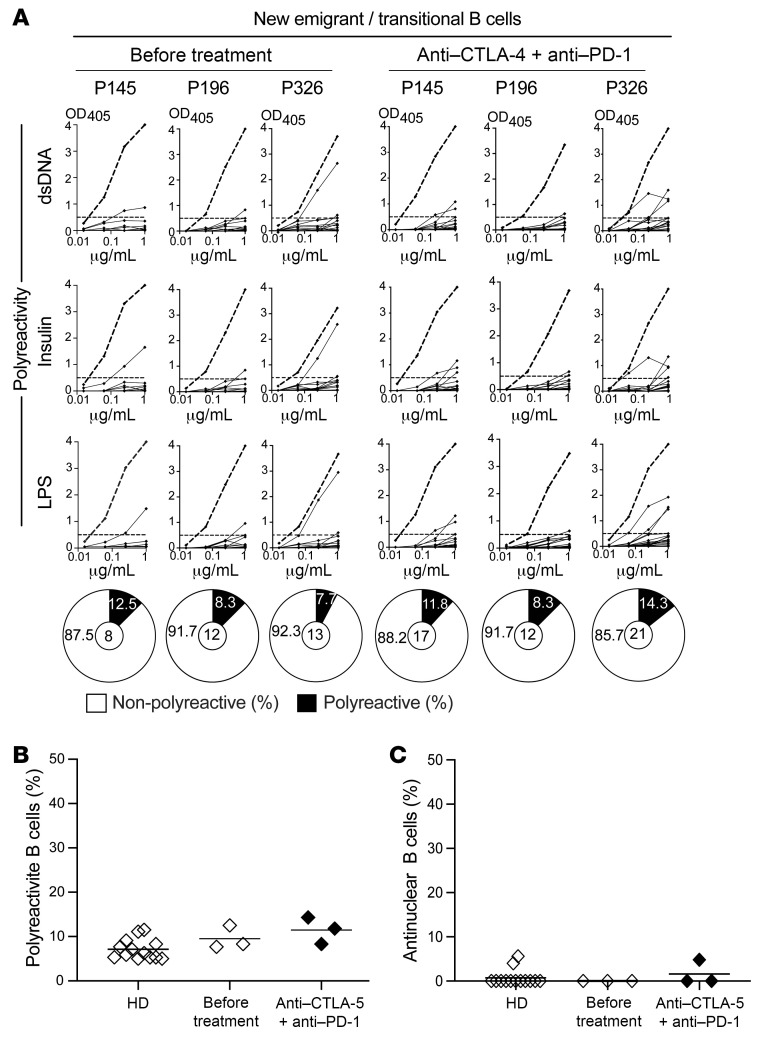
CPIs do not interfere with the establishment of central B cell tolerance. (**A**) Recombinant Abs cloned from single new emigrant/transitional B cells isolated from 3 cancer patients before and at 6 weeks after 2 rounds of anti–CTLA-4 and anti–PD-1 combination therapy were tested by ELISA for polyreactivity against dsDNA, insulin, and LPS. Dotted lines show the ED38 positive control. Horizontal lines show the cutoff OD_405_ for positive reactivity. For each individual, frequencies of nonpolyreactive (white area) and polyreactive (black area) clones are summarized in a pie chart below, with the total number of clones tested indicated in the centers. The frequencies of polyreactive and antinuclear reactive new emigrant/transitional B cells are summarized in **B** and **C**, respectively. Each symbol represents the reactivity data from each patient at the indicated time points determined from an average of *n* = 14 cloned recombinant antibodies. Averages are shown with a bar.

We first assessed the functionality of central B cell tolerance by comparing the frequencies of autoreactive clones in transitional B cells that recently emigrated from the bone marrow to the blood before and after anti–CTLA-4 + anti–PD-1 combination therapy. While we could not isolate single cells from the scarce new emigrant/transitional B cell compartment of cancer patient 134, we found that the proportions of polyreactive and antinuclear new emigrant/transitional B cells were low in the other 3 cancer patients before treatment, and similar to those of healthy donors previously analyzed (Figure 1). In addition, the frequencies of polyreactive and antinuclear clones in new emigrant/transitional B cells after anti–CTLA-4 and anti–PD-1 treatment remained as low as before treatment (Figure 1 and Supplemental Table 2). Hence, CPIs do not interfere with the establishment of central B cell tolerance.

**Figure 2:**
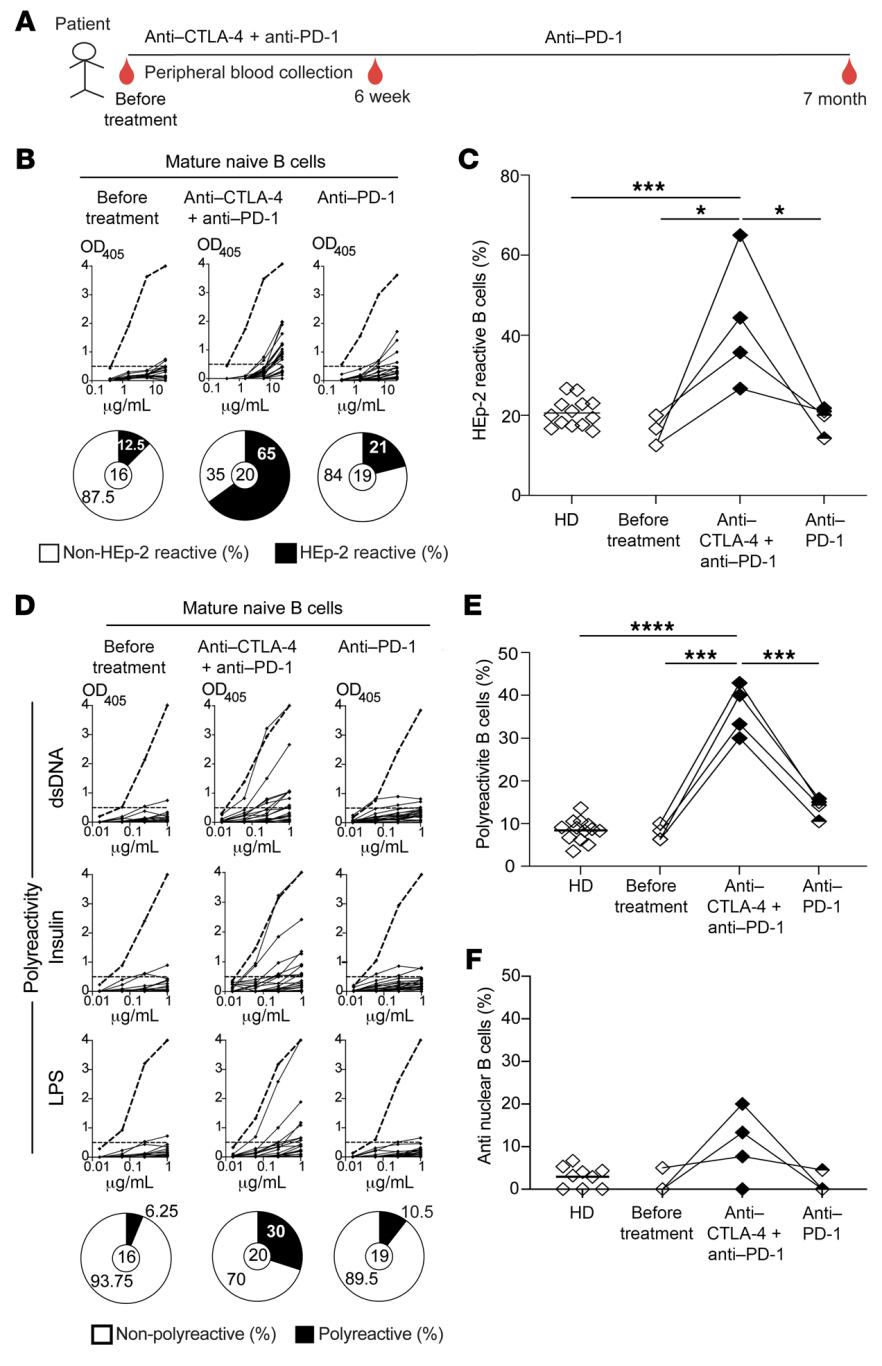
Anti–CTLA-4 and anti–PD-1 combination therapy results in the transient production of autoreactive mature naive B cells. (**A**) Schematic diagram summarizing treatments and time points for peripheral blood collection from patients with cancer. Recombinant Abs cloned from single mature naive B cells isolated from a representative cancer patient at different time points were tested by ELISA for anti-HEp-2 cell reactivity (**B**) and for polyreactivity defined by anti-dsDNA, anti-insulin and anti-LPS multireactivity (**D**). Dotted lines show the ED38 positive control. Horizontal lines show the cutoff OD_405_ for positive reactivity. For each individual, the frequency of nonreactive (white area) and reactive (black area) clones is summarized in a pie chart below, with the total number of clones tested indicated in the centers. The frequencies of HEp-2–reactive, polyreactive, and antinuclear-reactive mature naive B cells are summarized in **C**, **E**, and **F**. Each symbol represents an individual and data points were obtained from an average of *n* = 17 cloned recombinant antibodies. Averages are shown with a bar, and *P* values were determined by unpaired or paired Student’s *t* test. *P < 0.05, ***P < 0.001, ****P < 0.0001.

We have previously shown that a peripheral B cell tolerance checkpoint further counterselects autoreactive clones that escaped central B cell tolerance before they entered the mature naive B cell compartment (12). We therefore analyzed the frequencies of autoreactive mature naive B cells from 4 patients with cancer before treatment, at 6 weeks — which is after 2 cycles of anti–CTLA-4 and anti–PD-1 combination therapy — and at 7 months, when only treated with anti–PD-1 for almost 5 months (Figure 2A). We found that all 4 patients with cancer displayed low proportions of HEp-2-reactive, polyreactive, and antinuclear B cells before treatment, as in counterparts from healthy donors (Figure 2, B–F). In contrast, administration of anti–CTLA-4 and anti–PD-1 resulted in increased frequencies of HEp-2 reactive mature naive B cells, which represented 54% ± 12.6% compared with 17% ± 3.3% before treatment (2-way ANOVA, *P* value = 0.0175; Figure 2, B and C, Supplemental Figure 1A, and Supplemental Table 2). Peripheral B cell selection defects associated with the combination therapy were further evidenced by the elevated proportions of mature naive B cells that expressed polyreactive antibodies, which averaged 37.3% ± 7.6% compared with 8.5% ± 3.9% only 6 weeks earlier, when patients were untreated (2-way ANOVA, *P* value < 0.0001; Figure 2, D and E, Supplemental Figure 1B, and Supplemental Table 2), whereas the frequencies of clones expressing antinuclear antibodies with various nuclear staining patterns was only modestly increased by anti–CTLA-4 and anti–PD-1 mAbs (Figure 2F). All autoreactive B cell frequencies decreased back to normal low values at 7 months when anti–CTLA-4 treatment was terminated and the patients were only treated with anti–PD-1 (Figure 2, B–F, Supplemental Figure 1, and Supplemental Table 2). To further investigate the impact of anti–PD-1 mAb alone on the selection of developing autoreactive B cells, we assessed the frequencies of autoreactive clones in both new emigrant/transitional and mature naive B cells isolated from 2 additional patients with cancer only treated with anti–PD-1 for 24 and 28 months, respectively, and who did not develop major irAE. Unfortunately, we did not have pretreatment PBMC for comparison. We found that new emigrant/transitional and mature naive B cells from both patients with cancer who were only treated with anti–PD-1 and did not develop irAE contained low proportions of polyreactive and HEP-2–reactive clones as in healthy donors, thereby confirming that anti–PD-1 mAb does not alter either central or peripheral B cell tolerance checkpoints (Supplemental Figures 2 and 3). We conclude that treatment with anti–CTLA-4 and anti–PD-1 combination therapy induces a rapid accumulation of autoreactive B cells in the periphery, whereas anti–PD-1 mAb does not affect autoreactive B cell selection.

### Emerging autoreactive clones after anti–CTLA-4 and anti–PD-1 combination therapy may recognize self antigens expressed in tissues affected by irAEs.

**Figure 3:**
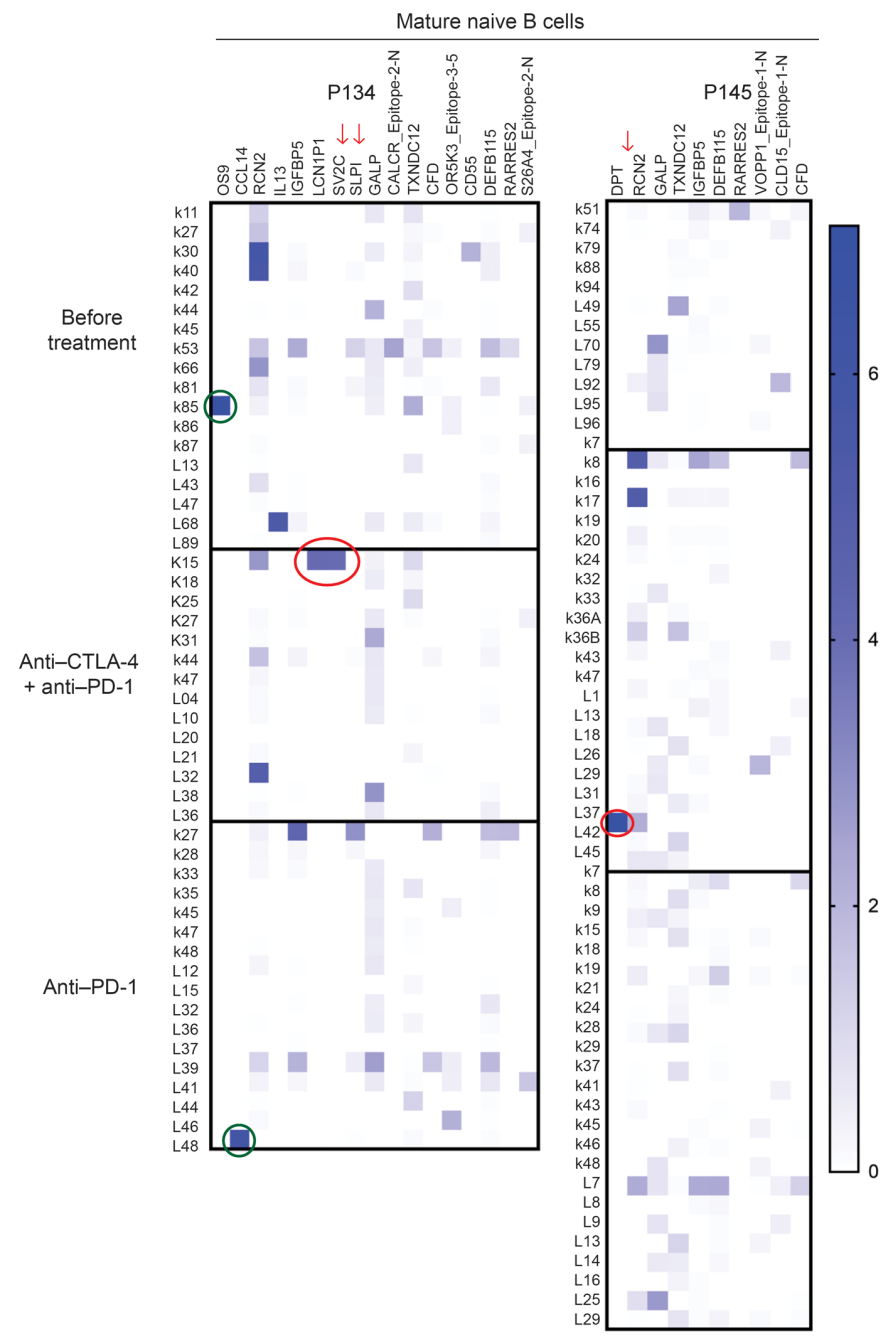
Autoreactive B cells induced by anti–CTLA-4 and anti–PD-1 combination therapy may recognize self antigens expressed in tissues affected by irAEs. The reactivity of recombinant Abs cloned from single mature naive B cells isolated from cancer patients 134 and 145 before treatment, at 6 weeks after 2 rounds of anti–CTLA-4 and anti–PD-1 combination therapy, and at 7 months when only treated with anti–PD-1 was screened using Rapid Extracellular Antigen Profiling (REAP). Heatmaps of REAP scores are represented. Each row corresponds to a recombinant antibody, and each column is a unique antigen. Red and green circles highlight detection of antibodies with unique specific reactivity from the anti–CTLA-4 + anti–PD-1 combination therapy or other time points, respectively. Score was artificially capped at 7 to aid visualization.

Since the transient production of autoreactive B cells induced by anti–CTLA-4 and anti–PD-1 combination therapy correlates with increased antitumor responses but also elevated risk of irAE, we intended to further characterize the reactivity of the recombinant antibodies cloned from single mature naive B cells isolated from patients with cancer at the 3 analyzed time points using Rapid Extracellular Antigen Profiling (REAP). This yeast-based technology can easily test antibody reactivity against up to almost 3,000 human antigens (13). We focused our analysis on recombinant antibodies that generated strong and specific REAP signals that were not shared by other recombinant antibodies (Figure 3 and Supplemental Figure 4). Specific hits were defined by the strong recognition of specific antigens that were only recognized by a single recombinant antibody. Remarkably, we found 2 clones with specific REAP signals at the 6-week time point after 2 rounds of anti–CTLA-4 and anti–PD-1 combination therapy that specifically recognized self antigens expressed in tissues that are targeted by irAE. Indeed, p145 6w λ37 recognized dermatopontin (DPT), which is expressed in skin, heart and pancreas, which all are organs attacked by the immune system following CPI treatment (Figure 3). In addition, recombinant antibody p134 6w κ15, which recognizes lipocaline 1-like protein 1 (LCN1P1), that is highly expressed in the thyroid, was cloned from patient p134, who developed thyroiditis (Figure 3). Clone p134 6w κ15 also bound to synaptic vesicle glycoprotein 2C (SV2C), which is expressed in the brain and endocrine glands, such as the pancreas and adrenal gland, which are organs also targeted by CPI-induced irAE (Figure 3). Of note, LCN1P1 and SV2C are also expressed in melanoma and other cancers such as breast cancers, respectively (Figure 3) (14). In contrast, protein OS-9 (OS9), which is a regulator of the hypoxic response, chemokine ligand 14 (CCL14), or ephrin B2 (EFNB2), which are recognized by clones from the pretreatment or treatment with anti–PD-1–only fractions are not expressed in irAE-targeted organs (Figure 3 and Supplemental Figure 4). REAP screening of patient’s serum samples at various time points did not identify an increase in secreted autoantibodies following CPI treatment, suggesting that the vast majority of autoreactive B cells induced by the combination therapy does not develop into autoantibody secreting cells (Supplemental Figure 5). Hence, anti–CTLA-4 and anti–PD-1 combination therapy allows the emergence of autoreactive B cells that recognize self antigens expressed in tissues that are targeted by irAE and also by tumor cells, which may promote antitumor responses.

### Single-cell RNA-seq analysis revealed an altered Treg compartment in cancer patients after anti–CTLA-4 and anti–PD-1 treatment.

**Figure 4:**
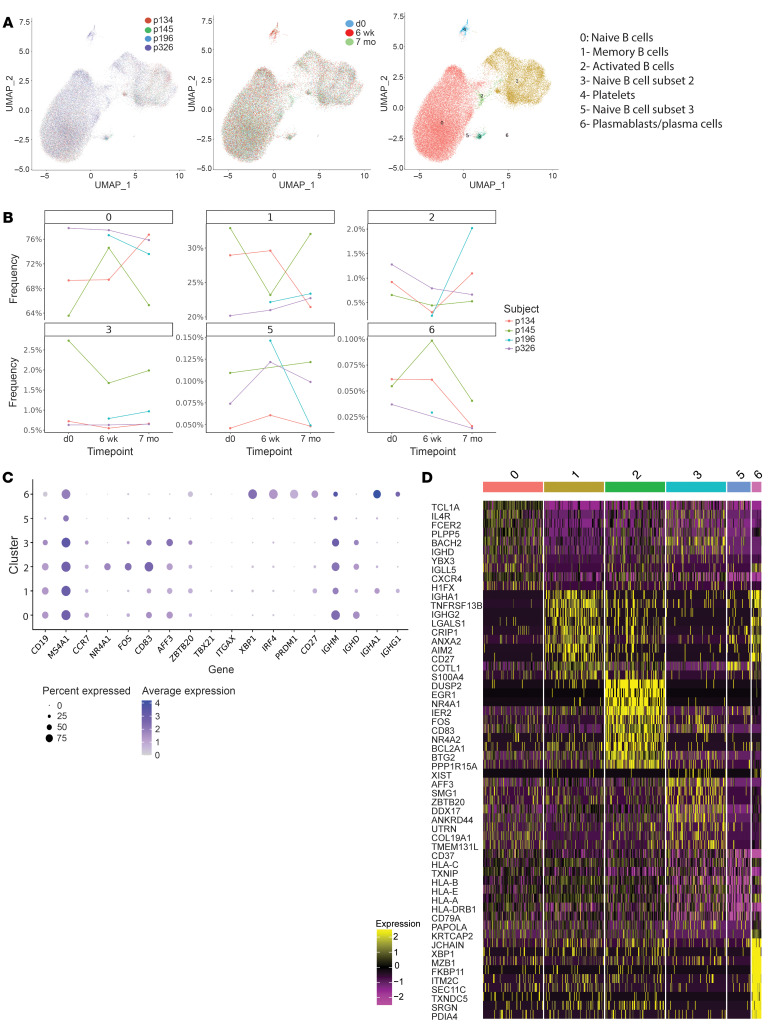
Single-cell analysis of circulating B cells in patients with cancer treated with CPIs. (**A**) Single-cell RNA-seq analysis of circulating B cells represented as UMAP graphs colored by each individual patient, time points, and clusters. Clusters were assigned to specific B cell subsets on the right. (**B**) Frequencies of each cluster at different time points are summarized separately. (**C**) Expression of phenotype-defining genes for each B cell cluster is represented. (**D**) Heatmap represents most differentially expressed genes for each cluster.

**Figure 5:**
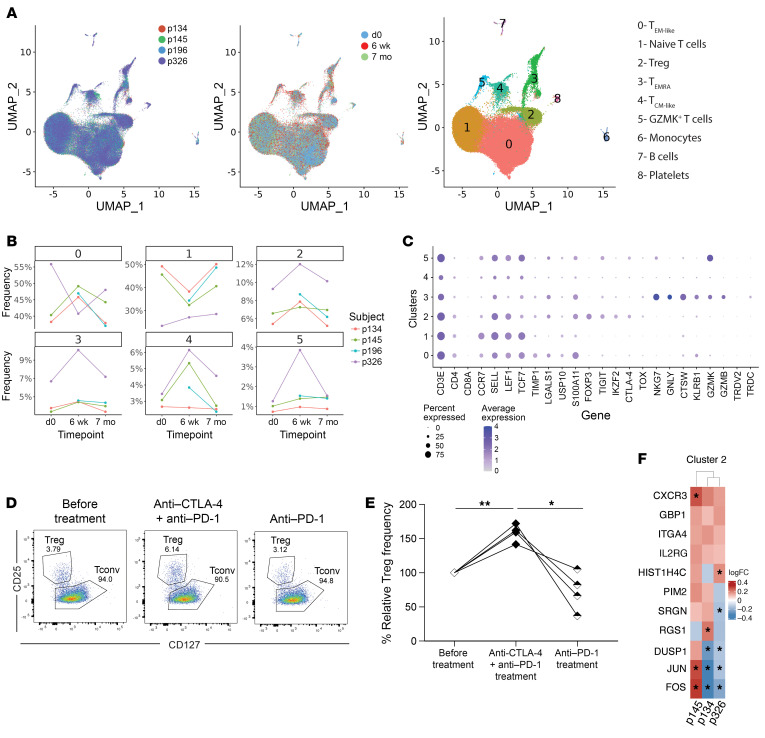
Single-cell analysis of circulating CD4^+^ T cell populations in patients with cancer treated with CPIs. (**A**) Single-cell RNA-seq analysis of circulating CD4^+^ T cells are represented as UMAP graphs colored by individual patients, time points, and clusters. (**B**) Frequencies of each cluster at different time points are summarized separately. (**C**) Expression of phenotype-defining genes for each cluster is represented. (**D**) Flow cytometry analysis of circulating gated regulatory T cells (Treg) and conventional non-Treg T cells (Tconv) is represented for each time point and percentages are summarized in **E** (paired Student’s *t* test, *P < 0.05, **P < 0.01). (**F**) Heatmap represents differentially expressed genes in Tregs after anti–CTLA-4 and anti–PD-1 combination therapy compared with counterparts before treatment and when on anti–PD-1 alone.

We used 5′ 10 × technology to investigate the impact of CPI on B cell and CD4^+^ T cell populations at single-cell resolution. We analyzed CD4^+^ T cells because we have recently demonstrated that censoring autoreactive B cells in the periphery involved the presentation by B cells of self antigens via MHC class II molecules to T cells and more specifically Tregs (11). B cell RNA-seq analysis from the 4 patients with cancer at the same 3 different time points revealed 6 different B cell clusters (Figure 4). While we observed a very heterogeneous naive B cell population, we could not identify any significant pattern common to all 4 patients and that may be associated with either the combination therapy or anti–PD-1 treatment alone (Figure 4). BCR sequencing analysis did not reveal any obvious expansion of specific clones, suggesting that the transient production of autoreactive B cells following anti–CTLA-4 and anti–PD-1 mAb injection is polyclonal (Supplemental Figure 6). In contrast, CD4^+^ T cell RNA-seq analysis revealed the specific expansion of cluster 2 in all 4 patients with cancer following anti–CTLA-4 and anti–PD-1 combination therapy, which corresponded to the time at which autoreactive B cells accumulated in the periphery (Figure 5, A and B). CD4^+^ T cells from cluster 2 are characterized by the expression of *FOXP3*, *TIGIT, IKZF2*, and *CTLA4* genes, which encode key molecules of the Treg lineage (Figure 5C). Flow cytometry analysis validated the enhanced frequencies of CD3^+^CD4^+^CD25^hi^CD127^lo^ Treg cells in all 4 patients with cancer at 6 weeks following 2 cycles of anti–CTLA-4 and anti–PD-1 mAbs compared with counterparts before treatment and at 7 months when only on anti–PD-1, whereas CD4^+^ and CD8^+^ T cell ratios and major CD4^+^ memory and effector T cell subsets were not affected by mAb treatments (Figure 5, D and E, and Supplemental Figure 7). In addition, Tregs from cluster 2 exhibited specific transcriptional differences induced by the combination therapy. The upregulation of *CXCR3*, *GBP1*, and *ITGA4* gene expression which are induced by IFN-γ and TNF-α, suggest that circulating Tregs after anti–CTLA-4 and anti–PD-1 mAb may have been exposed to an inflammatory milieu (Figure 5F). These Tregs also displayed enhanced expression of *PIM2* and *SRGN*, 2 genes characteristic of Tregs residing in nonlymphoid tissues, suggesting that the increased frequencies of Tregs at 6 weeks after combination therapy may result from a Treg migration from the tissues into the blood (Figure 5F) (15). In line with this hypothesis, decreased RGS1 expression favors T cell egress from the gut and CXCR3 promotes homing into inflamed tissues (Figure 5F) (16). Thus, our data show that the combination therapy alters Tregs that normally prevent the accumulation of autoreactive B cells in the periphery (11).

### CTLA-4 blockade is solely responsible for the production of autoreactive B cells in the periphery.

**Figure 6:**
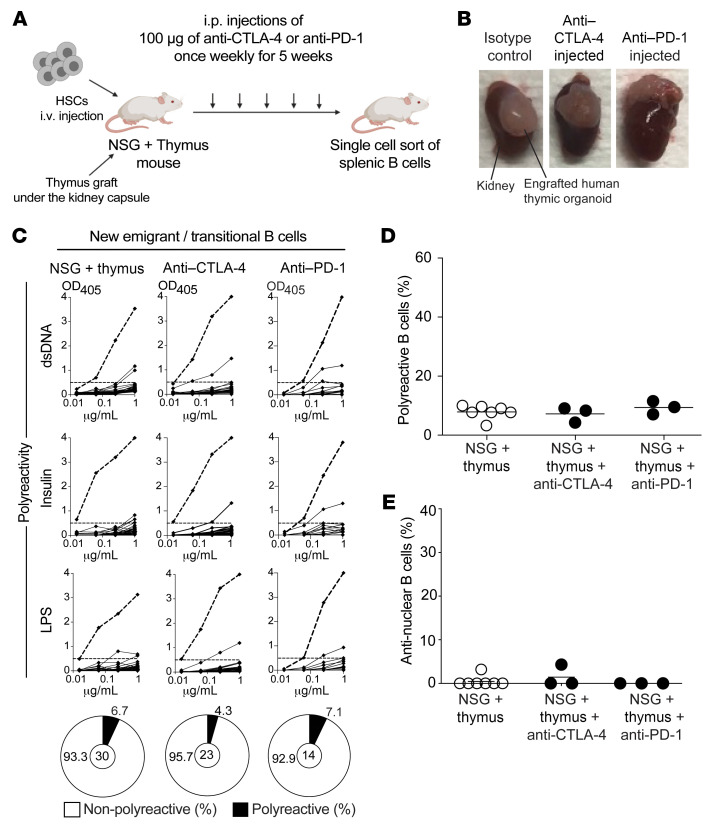
Anti–CTLA-4 and anti–PD-1 do not interfere with the establishment of central B cell tolerance. (**A**) Schematic diagram depicting the generation and injections of humanized mice. Fetal human CD34^+^ HSCs were injected i.v. into the adult NSG mice, along with surgical implantation of an autologous fetal thymic graft under the kidney capsule (NSG + thymus mouse model). After reconstitution, 100 μg of either anti–CTLA-4 or anti–PD-1 was injected intraperitoneally once a week for 5 weeks. (**B**) Representative pictures of the engrafted human thymic organoids of the indicated humanized mice. (**C**) Recombinant Abs cloned from single new emigrant/transitional B cells isolated from indicated humanized mouse were tested by ELISA for polyreactivity against dsDNA, insulin, and LPS reactivity. Dotted lines show the ED38 positive control. Horizontal lines show the cutoff OD_405_ for positive reactivity. For each mouse, frequencies of nonpolyreactive (white area) and polyreactive (black area) clones are summarized in a pie chart below, with the total number of clones tested indicated in the centers. The frequencies of polyreactive and antinuclear reactive new emigrant/transitional B cells are summarized in **D** and **E**, respectively. Each symbol represents the reactivity data for each humanized mouse determined from an average of *n* = 18 cloned recombinant antibodies. Averages are shown with a bar.

To determine whether anti–CTLA-4 is solely responsible for autoreactive B cell production, we analyzed the frequencies of autoreactive B cells in humanized mice that recapitulate both central and peripheral B cell tolerance checkpoints and that were injected with either anti–CTLA-4 or anti–PD-1 mAbs weekly for 4 weeks (Figure 6A). We recently reported that NOD-*scid* IL2Rγ^null^ (NSG) mice coengrafted with both human fetal HSCs and an autologous human fetal thymic graft under the kidney capsule displayed low frequencies of autoreactive clones both in transitional and mature naive B cells that were similar to B cell counterparts in healthy control blood (Figure 6B) (11). Humanized mice were injected intraperitoneally with anti–CTLA-4, anti–PD-1 mAbs or isotype controls when human CD45^+^ cells exceeded 50% of the lymphoid gate and when T cells did not express PD-1, which is induced during GvHD. hCD45^+^, CD3^+^ T, and CD19^+^ B cell ratio didn’t change after either anti–CTLA-4 or anti–PD-1 injections (data not shown). Injections of either anti–CTLA-4 or anti–PD-1 mAbs did not appear to affect thymocyte development in transplanted organoids, as illustrated by similar proportions of CD4^+^CD8^+^ double-positive and single CD4^+^ and CD8^+^ thymocytes between the different groups of humanized mice (Supplemental Figure 8A). The IgG1 isotype of the anti–CTLA-4 Ab used in our experiments did not appear to induce ADCC and/or ADCP because there was no evidence of Treg depletion in injected humanized mice (Supplemental Figure 8B). Indeed, proportions of human Tregs that express constitutive CTLA-4 and may express PD-1 in these humanized mice were not altered by either anti–CTLA-4 or anti–PD-1 injections (Supplemental Figure 8B). In addition, Tregs display normal low frequencies of HLA-DR^+^ cells, the majority of which express CCR7, 2 markers that validate their proper development in a human thymic environment (Supplemental Figure 8, C and D) (11). In addition, expression of LEF-1 and TCF1 transcription factors and BCL2 combined with low frequencies of Ki67^+^ Tregs were associated with effective Treg suppression function after either anti–CTLA-4 or anti–PD-1 injections, which further demonstrates the human thymic organoid origin of Tregs in these humanized mice and an absence of obvious Treg developmental abnormalities that may have been induced by these mAbs (Supplemental Figure 8, E–K) (11). Moreover, CTLA-4 expression in Tregs was not significantly altered by mAb injections, whereas anti–PD-1 decreased the detection of surface PD-1 expression on peripheral T cells and anti–CTLA-4 led to increased surface PD-1 expression due to T cell activation (Supplemental Figure 8, L–N).

**Figure 7:**
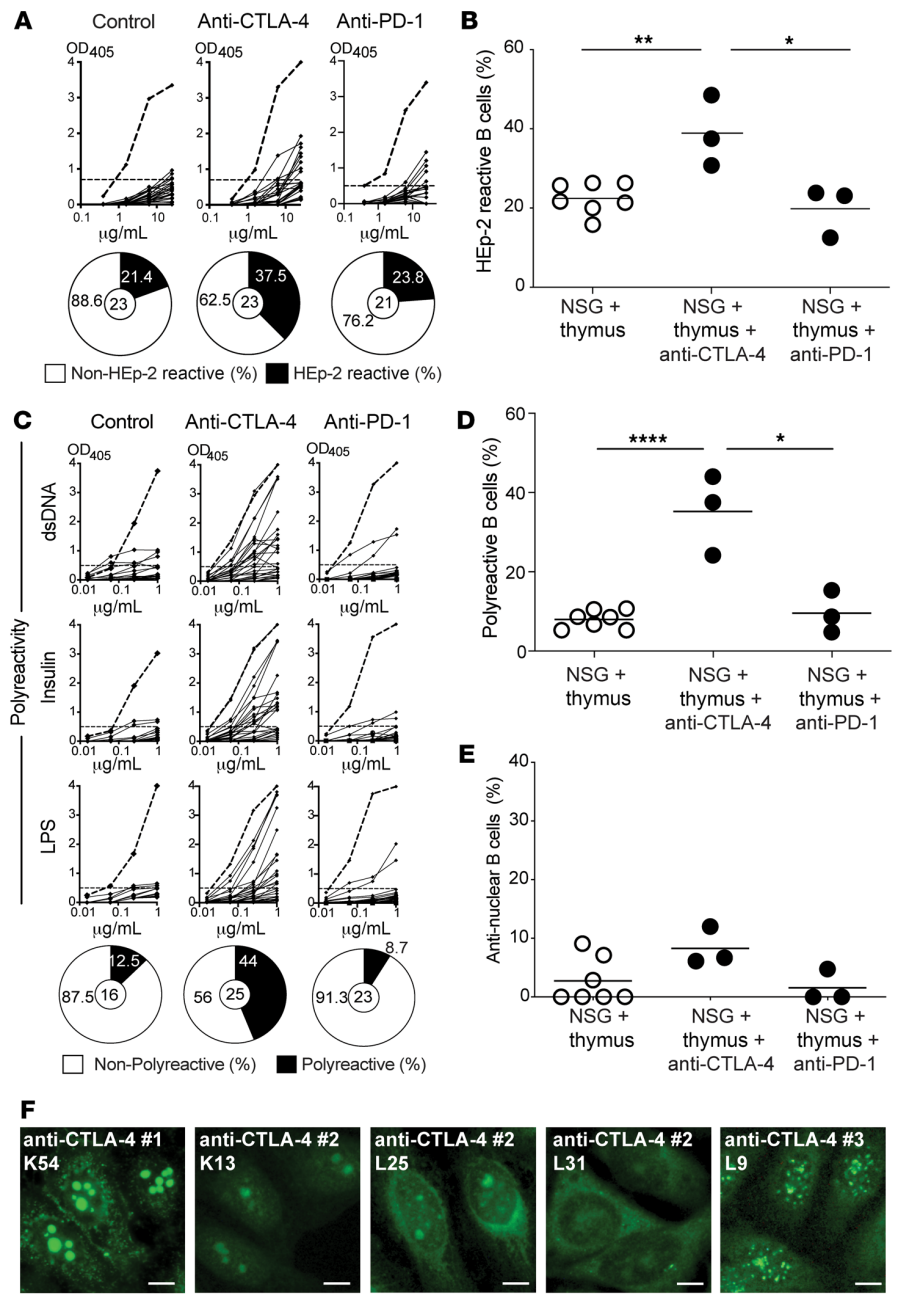
Anti–CTLA-4 injections induce an accumulation of autoreactive mature naive B cells. Recombinant Abs cloned from single mature naive B cells isolated from the indicated humanized mice were tested by ELISA for anti–HEp-2 cell reactivity (**A**) and for polyreactivity defined by anti-dsDNA, anti-insulin, and anti-LPS multireactivity (**C**). Dotted lines show the ED38 positive control. Horizontal lines show the cutoff OD_405_ for positive reactivity. For each humanized mouse, the frequency of nonreactive (white area) and reactive (black area) clones is summarized in a pie chart below, with the total number of clones tested indicated in the centers. The frequencies of HEp-2–reactive, polyreactive, and antinuclear-reactive mature naive B cells are summarized in **B**, **D**, and **E**. Each symbol represents a humanized mouse, and each data point was obtained from an average of *n* = 23 cloned recombinant antibodies. Averages are shown with a bar, and *P* values were determined by paired Student’s *t* test. *P < 0.05, **P < 0.01, ****P < 0.0001. (**F**) Representative nuclear staining patterns for recombinant antibodies cloned from single mature naive B cells.

We then investigated the frequencies of autoreactive B cells in new emigrant/transitional and mature naive B cells isolated from the spleens of humanized mice after 5 weeks of weekly 100 μg i.p. injections of anti–CTLA-4 or anti–PD-1 mAb. New emigrant/translational B cells from humanized mice injected with either anti–CTLA-4 or anti–PD-1 mAb contained low frequencies of polyreactive and antinuclear-reactive clones similar to control humanized mice, which confirmed that neither CTLA-4 nor PD-1 blockade interferes with the establishment of central B cell tolerance (Figure 6, A–E; Supplemental Figure 9, and Supplemental Table 2). In contrast, we found that anti–CTLA-4 mAb injections resulted in significantly elevated proportions of mature naive B cells in the spleen that expressed anti-HEp-2 reactive (2-way ANOVA; *P* = 0.0059), and polyreactive (2-way ANOVA; *P* = 0.0073) antibodies, whereas anti–PD-1 mAb did not alter the selection of autoreactive B cells (Figure 7, Supplemental Figure 10, and Supplemental Table 2). Consistent with this observation, we also identified elevated proportions of HEp-2 reactive and polyreactive mature naive B cells in historical frozen PBMC samples from a patient treated with anti–CTLA-4 monotherapy (Supplemental Figure 11 and Supplemental Table 2). Unfortunately, we could not analyze additional patients only treated with anti–CTLA-4 mAb because use of anti–CTLA-4 mAb as a single therapy is no longer common practice due to the lower anticancer activity and higher risk of irAE compared with anti–PD-1 treatment (1). We conclude that CTLA-4 blockade is solely responsible for the transient production of autoreactive B cells. These data also demonstrate that CTLA-4 plays an important role in preventing the expansion of autoreactive naive B cells in humans.

## Discussion

We reported herein an important and unexpected role for CTLA-4 in preventing the accumulation of developing autoreactive naive B cells, whereas PD-1 does not appear to be involved in the selection of the naive B cell repertoire. Indeed, all 5 out of 5 cancer patients who received anti–CTLA-4 treatment with or without combination with anti–PD-1 and 3 out of 3 humanized mice injected with anti–CTLA-4 displayed a specific accumulation of autoreactive mature naive B cells, thereby demonstrating that CTLA-4 blockade results in autoreactive B cell production. In contrast, none of the 6 cancer patients nor 3 humanized mice solely receiving anti–PD-1 mAb showed elevated frequencies of autoreactive B cells, demonstrating that PD-1 blockade does not interfere with the removal of developing autoreactive B cells. CTLA-4 blockade specifically antagonized the selection of autoreactive mature naive B cells in the periphery but did not interfere with the establishment of central B cell tolerance in the bone marrow, as illustrated by the low frequencies of autoreactive and antinuclear new emigrant/transitional B cells exiting the bone marrow after anti–CTLA-4 injections. We have previously reported the B cell–intrinsic regulation of the central B cell tolerance, which relies on proper BCR and TLR9 signaling (9, 10). Indeed, gene mutations affecting BCR or TLR9 function or the inhibition of either TLR9 or MYD88 expression in developing human B cells in humanized mice revealed the essential role of these molecules for the regulation of this early B cell counterselection checkpoint (9, 10, 17). Although CTLA-4 expression was identified in peritoneal autoreactive mouse B-1a B cells, it was not detected in bone marrow developing immature B cells (18). Hence, it was expected that CTLA-4 inhibition would not impact central B cell tolerance. Similarly, PD-1 expression on naive B cells is weak but our data exclude a putative role of PD-1 regulating BCR signaling during the early stages of B cell development because anti–PD-1 did not affect the negative selection of developing autoreactive B cells in the bone marrow.

Autoreactive B cells that escape central B cell tolerance are normally prevented from entering the mature naive B cell compartment, and we have suggested that this second peripheral B cell tolerance checkpoint removes self reactive clones that recognize autoantigens expressed in peripheral tissues (9, 19). Our studies also demonstrated that self-antigen presentation by MHC class II molecules on naive B cells and functional Tregs are essential for the silencing of peripheral autoreactive mature naive B cells (9, 11, 20). The involvement of Tregs was initially suggested by the analysis of immunodysregulation, polyendocrinopathy, enteropathy, X-linked (IPEX) patients who suffer from FOXP3 deficiency and showed elevated frequencies of circulating autoreactive mature naive B cells in the absence of functional Tregs (20). In addition, both decreased frequencies and impaired suppressive function in Tregs were associated with specific defects in the peripheral B cell tolerance checkpoint in patients with deficiency in autoimmune regulator (AIRE), lipopolysaccharide-responsive and beige-like anchor protein (LRBA), dedicator of cytokinesis 8 (DOCK8), or Wiskott-Aldrich syndrome protein (WASP), which suggests that the expansion of autoreactive B cells in the periphery is suppressed by Tregs (19, 21–23). The accumulation of autoreactive B cells following CTLA-4 blockade reveals now an essential role for CTLA-4 in regulating this peripheral B cell selection step. Of note, autoreactive B cells that specifically escaped the peripheral B cell tolerance checkpoint rarely contained antinuclear clones that are counterselected during central B cell tolerance (9, 10). scRNA-seq analysis of B cells revealed an absence of proliferation following treatment by CPI, suggesting that the emergence of autoreactive B cells in the blood of patients with cancer 6 weeks after the combination therapy is not due to the expansion of preexisting autoreactive clones. We therefore favor the scenario in which CTLA-4 blockade inhibits the negative selection of newly generated B cells that recognize peripheral tissue antigens and that are continuously produced by the bone marrow. Since CTLA-4 is expressed constitutively by Tregs and inhibits the expression of CD80/CD86 expression via transendocytosis, anti–CTLA-4 mAb may antagonize this tolerogenic mechanism and allow instead the interaction of activated autoreactive B cells with T cells that provide survival signals and prevent their silencing (24–26). Indeed, mouse models in which CD80 or CD86 were overexpressed on B cells failed to eliminate autoreactive B cells via a Fas-dependent mechanism during B cell–T cell interactions (27, 28). It is unclear where the peripheral counterselection of autoreactive B cells occurs. A previous study suggested that human transitional B cells migrate to the gut, where autoreactive clones may be eliminated (29). Our data support this hypothesis in that the increase in autoreactive B cells following combined CTLA-4 and PD-1 blockade in patients with cancer was associated with elevated proportions of blood Tregs with decreased *RGS1* expression, which favors T cell egress from the gut (30). In addition, these peripheral Tregs expressed genes characteristic of tissue-resident Tregs, suggesting a dysregulation of Treg homeostasis in the tissues and gut where the peripheral B cell tolerance checkpoint may take place (15, 30, 31). While CTLA-4 blockade may impede the important tolerogenic function reported for CTLA-4 in Tregs, the increase in *PIM2* that encodes a kinase that phosphorylates Foxp3 and decreased *RGS1* expression were both reported to diminish Treg immunosuppressive function, which suggests that combined CTLA-4 and PD-1 blockade may lead to an inability for Tregs to prevent the expansion of autoreactive naive B cells (30–32). As a consequence, REAP analysis revealed that the repertoire of mature naive B cells after CTLA-4 blockade contains self-reactive clones that recognize self antigens expressed in endocrine glands and other peripheral tissues targeted by irAEs. This induced autoreactive repertoire is similar to those of patients with autoimmune diseases, and autoreactive B cells may induce the development of irAEs frequently associated with anti–CTLA-4 treatment by presenting self antigens through their MHC class II to T cells (9). Of note, we failed to detect the secretion of autoantibodies in the serum of patients after CPI treatment, suggesting that some peripheral tolerance mechanisms, such as the TLR9-mediated inhibition of autoantibody secretion, likely remain functional (33, 34).

The use of anti-B cell therapy, which showed clinical efficacy in several patients with autoimmune diseases who also displayed elevated frequencies of autoreactive naive B cells in their blood may prevent the development of irAEs in CPI-treated cancer patients if given either prior to or shortly after administration of anti–CTLA-4 mAb. A potential role of autoreactive B cells in promoting antitumor responses may represent an important limitation to anti-B cell therapy as preventive treatment of irAEs. Indeed, the broadening of the peripheral BCR repertoire by anti–CTLA-4 may not only include self-reactive clones but potentially also B cells that may express tumor self- or neoantigen-reactive BCRs. Consistent with this scenario, B cell infiltration and tertiary lymphoid structures in the tumor microenvironment of patients treated with CPI treatment positively correlate with improved antitumor responses and patient survival (35–37). B cells that recognize tumor antigens may promote antitumor immunity via the development of T follicular helper cells and their production of IL-21, which enhances CD8^+^ T cell effector functions (38).

In summary, we report that intact CTLA-4 function is required for the removal of developing autoreactive mature naive B cells in the periphery, whereas anti–PD-1 mAb does not interfere with autoreactive B cell selection. The emergence of autoreactive B cells following CTLA-4 blockade includes clones that may recognize self antigens expressed in tissues affected by irAEs but also tumor neo- or self antigens and thereby promote antitumor responses. Additional studies are warranted to investigate the reactivity of B cells infiltrating tumors and determine the antigens they recognize as well as the potential proper timing for anti–B cell therapy to eliminate the autoimmunity initiating function of autoreactive B cells that emerge after CPI treatment.

## Methods

### Sex as a biological variable.

Our study examined male and female patients and similar findings are reported for both sexes. Human fetal tissues from both sexes were also investigated in the study (Supplemental Table 3).

### Patients and fetal tissues.

Blood samples were collected at the Yale School of Medicine. Characteristics of cancer patients and their disease profiles are summarized in Supplemental Table 1. Fetal samples with chromosomal abnormalities and those that tested positive for the *PTPN22* 1858T risk allele were excluded since the presence of this polymorphism is sufficient to induce the development of autoreactive B cells (39). Age and sex of each fetus used in the study are documented in Supplemental Table 3.

### Humanized mice.

Immunodeficient NOD*-scid* common γ chain–deficient (NSG) mice were purchased from The Jackson Laboratory (stock no. 005557). Ten-week-old female NSG mice were used in thymic transplantation surgeries to generate NSG + thymus humanized mice (11). HSCs and thymic tissues from both male and female fetuses were used in the generation of NSG + thymus humanized mice. Human CD34^+^ cells were purified from fetal liver and bone marrow by density-gradient centrifugation followed by positive immunomagnetic selection with anti–human CD34 microbeads (Miltenyi Biotec). To generate NSG + thymus humanized mice, 9- to 11-week-old female NSG mice were conditioned with 2.5 Gy total body irradiation (x-ray irradiation with the Precision X-Ray X-RAD 320 irradiator) the day before surgery. Human fetal thymus fragments measuring approximately 1 mm^3^ were then implanted under the kidney capsule of the recipient mice. CD34^+^ fetal HSCs (200,000–300,000 cells) from the same donor were injected i.v. shortly after the surgery. All mice were used for experiments 10–12 weeks after HSC transplantation. NSG + thymus humanized mice were injected intraperitoneally with 100 μg of either anti–CTLA-4 (mouse IgG1, clone L3D10, which blocks CTLA-4/CD80 interaction, Biolegend), Nivolumab anti–PD-1 Abs, or a combination of mouse IgG1 (clone G3A1, Cell Signaling Technology) and IgG4 (clone QA16A15, Biolegend) isotype controls once a week for 5 weeks. All animals were treated, and experiments were conducted in accordance with the Yale institutional review guidelines on treatment of experimental animals.

### B cell and CD4^+^ T cell staining and sort.

B cells were purified from peripheral blood mononuclear cells (PBMCs) of patients using CD20 beads (Miltenyi) or from the spleen from humanized mice by positive selection using CD19 magnetic beads (Miltenyi). Enriched B cells were stained with antibodies listed in Supplemental Table 4. CD19^+^CD21^–/lo^CD10^+^IgM^hi^CD27^–^ new emigrant/transitional B cells and CD19^+^CD21^+^CD10^–^IgM^+^CD27^–^ mature naive B cells from the blood of patients or the spleen of humanized mice were sorted on a FACSAria sorter (Becton Dickinson) into 96-well PCR plates as single cells or batch-sorted into 5-mL round bottom polystyrene test tubes. The samples were immediately frozen on dry ice. The gating strategies to sort these B cell subsets are provided in Supplemental Figure 1. CD4^+^ T cells were enriched from the non–B cell fractions using the EasySep Human CD4^+^ T cell enrichment kit (STEMCELL Technologies) and stained with Abs against human CD3, CD4, CD25, and CD127 (also listed in Supplemental Table 4). CD3^+^CD4^+^CD25^hi^CD127^lo^ Tregs and CD3^+^CD4^+^CD25^–/lo^ CD127^+^ T conventional cells were then batch sorted for in vitro suppression assays.

### B cell and Treg immunophenotyping.

After CD19 microbead magnetic separation, non–B cell fractions from HDs or humanized mice were stained with Abs against the Treg markers CD3, CD4, CD25, and CD127 as well as the surface markers of interest CXCR3, CCR5 ICOS, HLA-DR, CCR7, and CTLA-4. Abs against intracellular markers of interest including FOXP3, TCF1, LEF1, BCL2, and Ki67 were added after fixation and permeabilization of T cells (Invitrogen, Thermo Fisher Scientific). All mAbs used are listed in Supplemental Table 4. Flow cytometry was performed on a BD LSR II flow cytometer, and the data were analyzed with FlowJo software.

### cDNA, RT-PCR, antibody production and purification.

RNA from single B cells was reverse transcribed in the original 96-well plate in 12.5-μL reactions containing 100 U of Superscript II RT (Gibco BRL) for 45 min at 42°C. RT-PCR reactions, primer sequences, cloning strategy, expression vectors, antibody expression, and purification were carried out as previously described (12).

### Antibody sequence analysis.

Immunoglobulin sequences and mutation status were determined using Ig BLAST comparison with GenBank using the National Center for Biotechnology Information IgBlast server (http://www.ncbi.nlm.nih.gov/igblast/). Heavy chain complementarity determining region 3 was defined as the interval between amino acid at position 94 in the *V_H_* framework 3 and the conserved tryptophan at position 103 in the Ig heavy chain joining gene (*J_H_*) segment. Antibody sequences and reactivity are shown in Supplemental Table 4.

### ELISAs and indirect immunofluorescence staining.

Antibody concentrations and reactivity were measured as previously described (12). Highly polyreactive ED38 was used as positive control in HEp-2–reactivity and polyreactivity ELISAs. Antibodies were considered polyreactive when they recognized all 3 analyzed antigens: double stranded DNA (dsDNA), insulin, and lipopolysaccharide (LPS). The cut off used to define positivity of OD405 corresponds to twice the average OD405 ELISA value of all tested clones and is approximatively 0.5. For indirect immunofluorescence assays, HEp-2 cell-coated slides (Bion Enterprises) were incubated in a moist chamber at room temperature with purified recombinant antibodies at 50–100 μg/mL and detected with FITC-conjugated goat anti-human IgG.

### REAP Assay.

REAP assay was performed to test the reactivity of recombinant antibodies cloned from single mature naive B cells of patients with cancer (*n* = 9) and in their serum after IgG antibodies were isolated using protein G magnetic resin (Lytic Solutions) (13). In brief, after yeast library selection with 10 μg patient-derived antibody, DNA was extracted using Zymoprep-96 Yeast Plasmid Miniprep kits or Zymoprep Yeast Plasmid Miniprep II kits (Zymo Research). After 2 PCR rounds with 1 μL plasmid DNA and run on a 1% agarose gel, DNA (NGS library) was extracted from the band corresponding to 257 base pairs by QIAquick Gel Extraction Kit (Qiagen). NGS library was sequenced using an Illumina NextSeq 500 and NextSeq 500/550 75 cycle High Output Kit v2.5 with 75 base pair single-end sequencing according to standard manufacturer protocols. A minimum of 50,000 reads per sample was collected and the preselection library was sampled at 10-times greater depth than other samples. REAP data analysis was performed as previously described (13).

### Sample preparation for single-cell RNA-seq analysis.

Single-cell suspension of CD20^+^ B cells and CD4^–^ T cells from patients with cancer were prepared in 0.04% PBS-BSA buffer at 1,000 cells per μL concentration in precoated 1.5 mL Eppendorf tubes. The viability of the cells was checked with trypan blue and was more than 90% for all samples. Samples were sent to Yale YCGA for isolation and sequencing of RNA using 5′ 10× Chromium Single Cell and VDJ sequencing technology.

### Processing of 10× Genomics single-cell 5′ gene expression data.

To generate count matrices, barcode assignments, and feature calls, we used the Cell Ranger count subcommand. Seurat v4.3.0 was used for gene expression analysis (40). We removed cells with fewer than 400 transcripts or with mitochondrial content of more than 15% of all transcripts. We library-normalized and log-transformed the UMI counts and selected the top 2,000 variable genes using the “FindVariableFeatures” function with the “vst” option. We removed immunoglobulin and T cell receptor–related genes from the list of highly variable genes so that their properties could be analyzed independently of cell type annotation. We centered and scaled the data, ran principal component analysis and took the first 50 principal components. We removed the batch effect between samples using harmony on the principal components (41). The corrected principal components were used to generate the UMAP embeddings and perform clustering using Seurat “FindClusters” function with a resolution of 0.1.

### Processing of 10× Genomics single-cell TCR/BCR reads.

We annotated TCR and BCR data using the Cell Ranger vdj command. We removed sequences with nonproductive arrangements and filtered for cells with exactly one TCR-β, or BCR heavy chain sequence. We further processed the BCR data using Immcantation suite (42). B cell clones were inferred based on heavy chain sequences using hierarchical clustering with single-linkage for each individual. Specifically, sequences were partitioned based on common V and J gene annotations and junction lengths. Within each partition, sequences whose junction sequences were within 0.09 normalized Hamming distances between each other were clustered as heavy chain clones. The cutoff was chosen based on a visual inspection of the bimodal distance-to-nearest distribution of hamming distances. The heavy chain clones were further divided based on light chain gene usage as the final clones.

### Treg suppression assays.

CD3^+^CD4^+^CD25^–/lo^CD127^+^ Tconv cells were labeled with CellTrace CFSE (Invitrogen, Thermo Fisher Scientific). Cocultures of Tregs and Tconv cells at a 1:1 ratio were stimulated with the Treg Suppression Inspector Human kit (Miltenyi Biotec), which contains anti-CD2/anti-CD3/anti-CD28, at a 1 bead/1 cell ratio. The proliferation of viable Tconv cells was analyzed by CFSE dilution using flow cytometry 3.5 days after stimulation.

### Statistics.

Statistical analysis was performed using GraphPad Prism (version 6 or 7.03; GraphPad). Differences between groups of research participants were analyzed for statistical significance with 2-way ANOVA statistical analysis for repeated measures in the same patients or humanized mice engrafted with the same fetal tissues. Individual differences induced by the injections of mAbs in patients or humanized mice were evaluated using either unpaired Student’s *t* test when comparing healthy donors with patients or paired Student’s *t* test for internal comparison of various treatments in patients or humanized mice. Multiple group comparisons were corrected for statistical significance with the Bonferroni-Dunn method. A *P* value of ≤0.05 was considered significant.

### Study approval.

Informed consent was obtained from all patients before participation. Tissues from 105- to 135-day-old human fetuses, including bone marrow, spleen, liver, and thymus tissues, were obtained from the Birth Defects Research Laboratory at the University of Washington with ethics board approval and maternal written consent. This study was approved and performed in accordance with ethical and legal guidelines of the Yale University institutional review board (protocol number 0906005336).

### Data and code availability.

Values underlying the data provided in our study are available in the Supporting Data Values file. Sequencing data have been deposited at the NCBI’s Gene Expression Omnibus (GEO) database (GEO GSE307261).

## Author contributions

EÇ, HMK, KCH, and EM conceptualized the study and designed the methodology. EÇ, AM, CRVP, DB, JMB, RG, SY, LC, FRD, and CM performed studies. MW analyzed the single-cell RNA-seq data, supervised by YK; YD performed REAP assay supervised by AMR; ALP, AB, HMK, and KCH recruited patients. EÇ and EM wrote the original draft of the manuscript. EM supervised the work.

## Funding support

This work is the result of NIH funding, in whole or in part, and is subject to the NIH Public Access Policy. Through acceptance of this federal funding, the NIH has been given a right to make the work publicly available in PubMed Central.

- NIH grant CA227473 to KCH, HMK, and EM.

## Supplementary Material

Supplemental data

Supplemental table 2

Supporting data values

## Version 1 09/30/2025

In-Press Preview

## Version 2 11/17/2025

Electronic publication
