# Supplementary material for: CTLA-4 blockade shifts the B cell repertoire toward autoimmunity
Source: J Clin Invest. 2025 Sep 30;135(22):e189074. doi: 10.1172/JCI189074 (PMC12618075; doi:10.1172/JCI189074)
Supplement: Supplemental data [file jci-135-189074-s143.pdf]

Supplementary Materials for

CTLA-4 Blockade Shifts the B Cell Repertoire Towards Autoimmunity

Elif Çakan, Meng Wang, Yile Dai, Adrien Mirouse, Clarence Rachel Villanueva-Pachas, Delphine Bouis, Joshua M. Boeckers, Ruchi Gera, Sally Yraita, Leslie Clapp, Ana Luisa Perdigoto, Fabien R. Delmotte, Christopher Massad, Antonietta Bacchiocchi, Aaron Ring, Yuval Kluger, Harriet M. Kluger, Kevan C. Herold and Eric Meffre

Correspondence to: emeffre@stanford.edu

This PDF file includes:

Supplemental Figures 1 to 11

Other Supplemental Materials for this manuscript include the following:

Supplemental Table 1. Characteristics of melanoma patients treated with CPIs.
Supplemental Table 2 (Excel file). Repertoire and reactivity of recombinant antibodies cloned from single new emigrant/transitional and mature naïve B cells from cancer patients and humanized mice.
Supplemental Tables 3. List of human fetal samples used in the study.
Supplemental Tables 4. Antibody reagents, dye and dilutions used in the study.

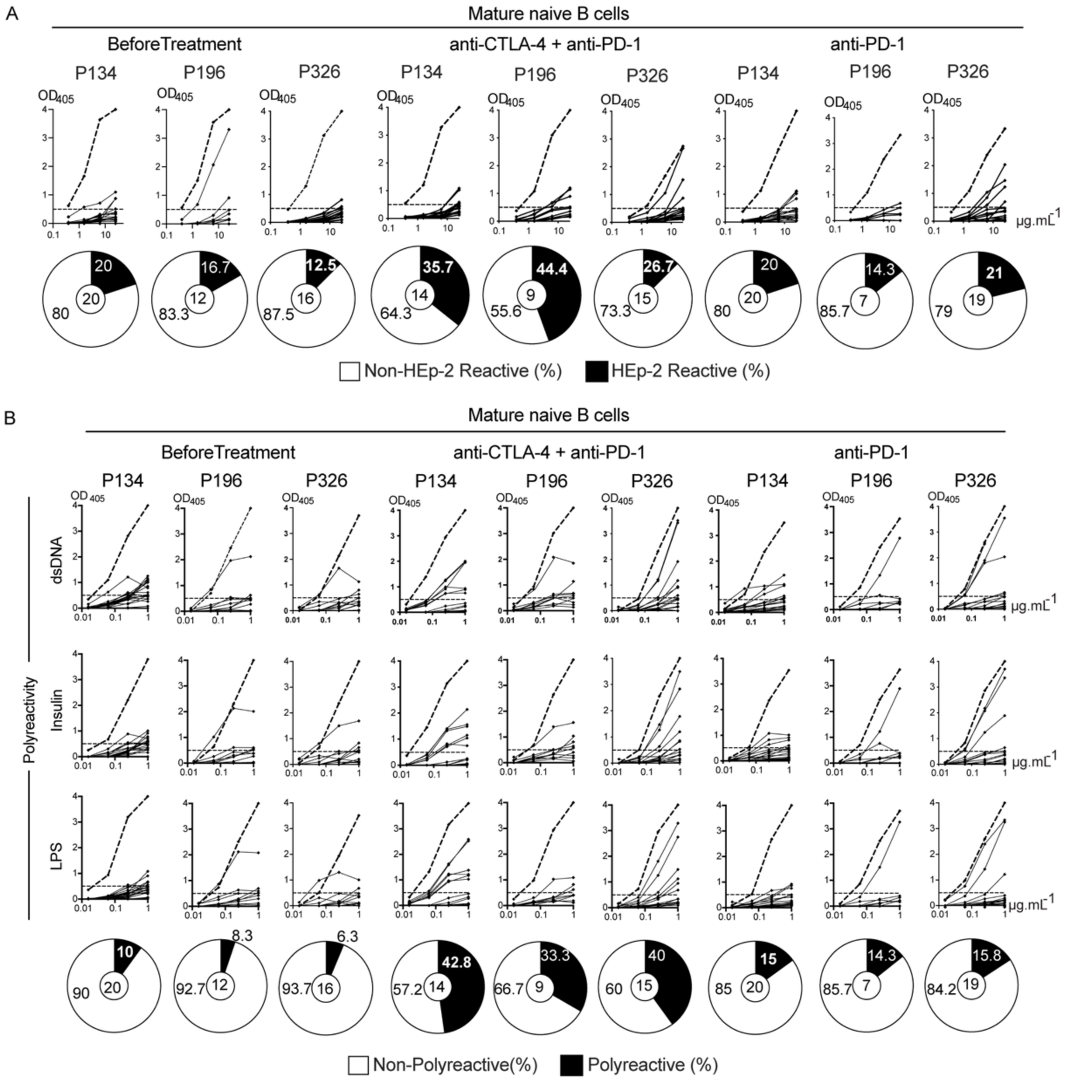

Supplemental Figure 1. Anti-CTLA-4 and anti-PD-1 combination therapy induces an accumulation of autoreactive mature naïve B cells in cancer patients. Recombinant Abs cloned from single mature naïve B cells isolated from the indicated cancer patients were tested by ELISA for anti–HEp-2 cell reactivity (**A)** and polyreactivity tested using dsDNA, insulin and LPS antigens (**B**). Dotted lines show the ED38 positive control. Horizontal lines show the cutoff OD_{405} for positive reactivity. For each individual, frequencies of non-reactive (white area) and reactive (black area) clones are summarized in a pie chart below, with the total number of clones tested indicated in the centers.

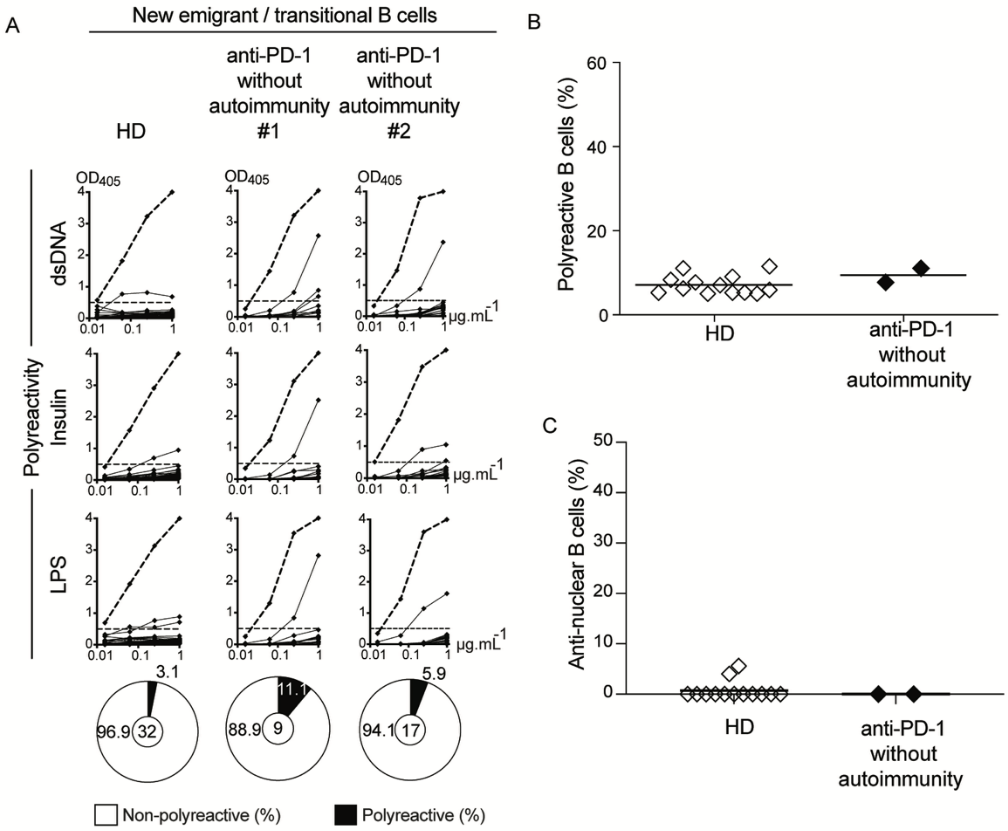

Supplemental Figure 2. Anti-PD-1 does not interfere with central B-cell tolerance. (**A**) Recombinant Abs cloned from single new emigrant/transitional B cells isolated from two cancer patients who did not suffer from irAEs after anti-PD-1 treatment were tested by ELISA for polyreactivity against dsDNA, insulin and LPS. Dotted lines show the ED38 positive control. Horizontal lines show the cutoff OD_{405} for positive reactivity. For each patient, the frequencies of non-polyreactive (white area) and polyreactive (black area) clones are summarized in a pie chart below, with the total number of clones tested indicated in the centers. The frequencies of polyreactive and anti-nuclear reactive new emigrant/transitional B cells are summarized in (**B**) and (**C**), respectively. Each symbol represents the reactivity data from each patient. Averages are shown with a bar.

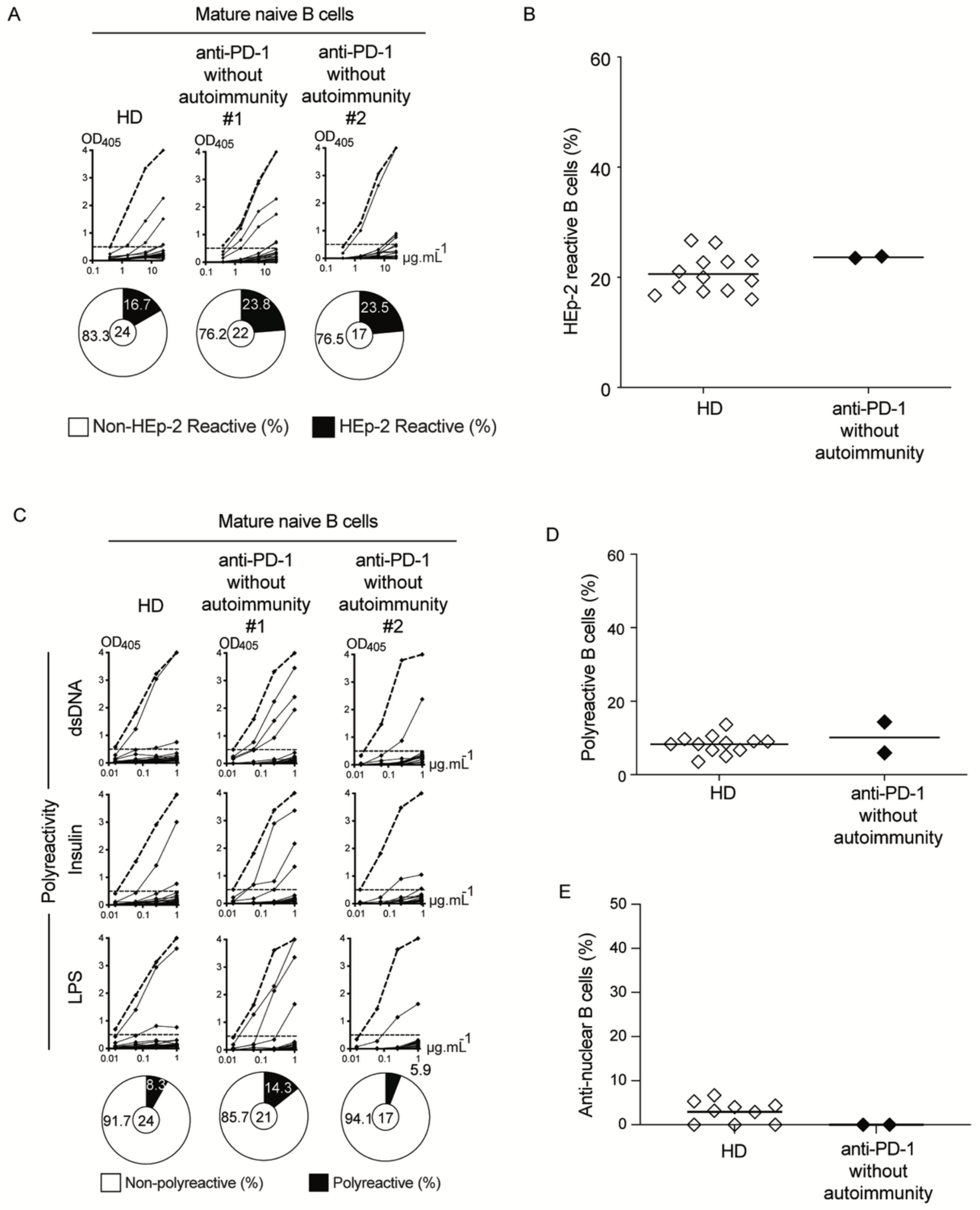

Supplemental Figure 3. Anti-PD-1 does not induce the production of autoreactive mature naïve B cells. Recombinant Abs cloned from single mature naïve B cells isolated from two cancer patients who did not suffer from irAEs after anti-PD-1 treatment were tested by ELISA for anti-HEp-2 cell reactivity (**A**) and for polyreactivity defined by anti-dsDNA, anti-insulin and anti-LPS multi-reactivity (**C**). Dotted lines show the ED38 positive control. Horizontal lines show the cutoff OD_{405} for positive reactivity. For each individual, the frequency of non-reactive (white area) and reactive (black area) clones is summarized in a pie chart below, with the total number of clones tested indicated in the centers. The frequencies of HEp-2–reactive, polyreactive, and anti-nuclear-reactive mature naive B cells are summarized in (**B**)**,** (**D**)**,** and (**E**). Each symbol represents an individual. Averages are shown with a bar.

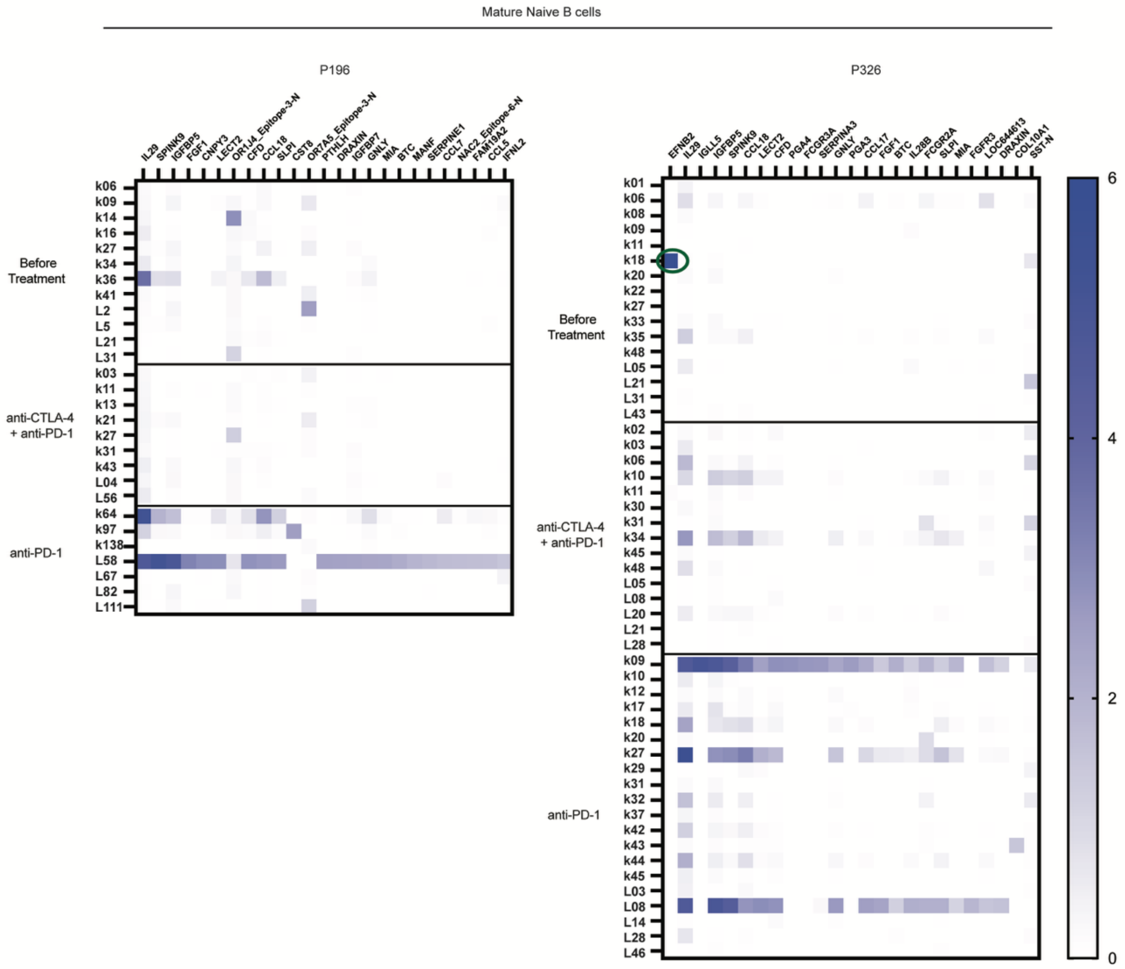

Supplemental Figure 4. Heatmap of recombinant antibody REAP scores. The reactivity of recombinant Abs cloned from single mature naïve B cells isolated from cancer patients 196 and 326 before treatment, at six weeks after two rounds of anti-CTLA-4 and anti-PD-1 combination therapy and at 7 months when only treated with anti-PD-1 was screened using Rapid Extracellular Antigen Profiling (REAP). Each row corresponds to a recombinant antibody and each column is a unique antigen. The green circle highlights detection of an antibody with unique specific reactivity from the before treatment fraction of cancer patient 326. Score was artificially capped at 6 to aid visualization.

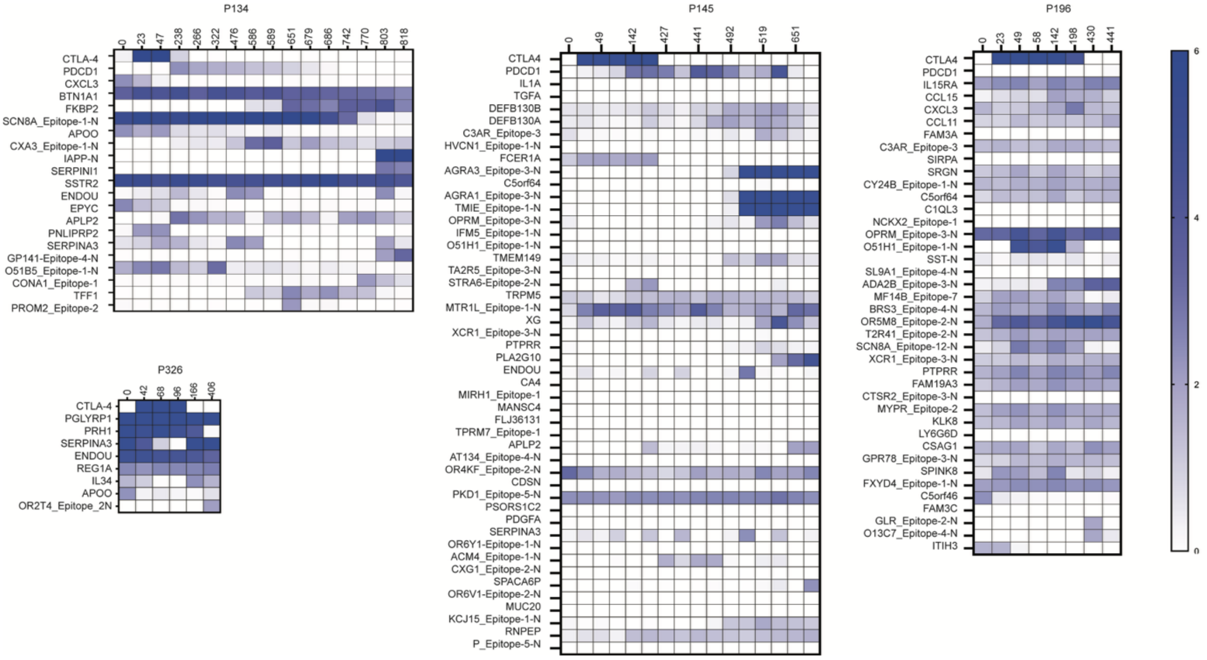

Supplemental Figure 5. Heatmap of REAP scores for serum antibodies from cancer patients. The reactivity of serum antibodies from cancer patients 134, 145, 196 and 326 before treatment and at various days post CPI treatment was screened using Rapid Extracellular Antigen Profiling (REAP). Each row is a specific antigen, and each column is a serum sample from the indicated cancer patients collected at various days post CPI treatment. Score was artificially capped at 6 to aid visualization.

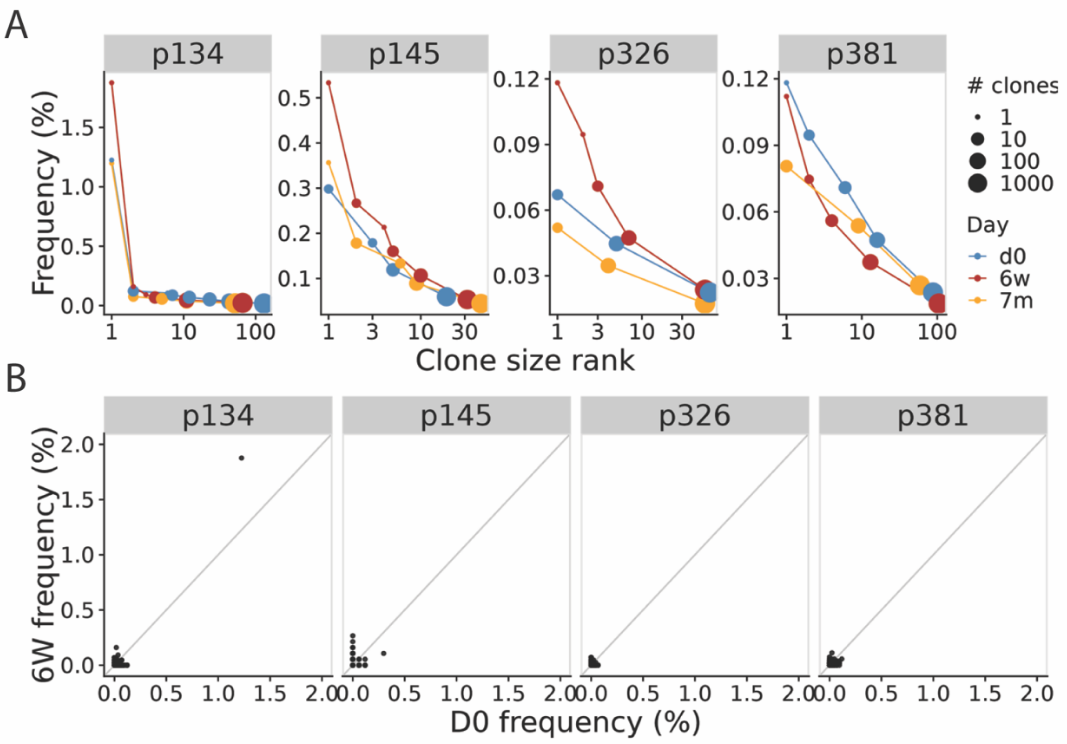

Supplemental Figure 6. **Absence of obvious B cell clonal expansion following treatment with anti-CTLA-4 and anti-PD-1 mAbs**. (**A**) Rank-abundance plot for B cell clone size distribution. The size of the dots indicates the number of clones. The x-axis indicates the rank of the clone size, with the rank of 1 being the largest. The y-axis indicates the relative abundance of the clone within the sample. The color indicates the time points. (**B**) Scatter plot of clone frequencies at day 0 and week 6. Fisher's exact test with FDR correction was used to identify significantly expanded clones and no clones were found to significantly expand at week 6.

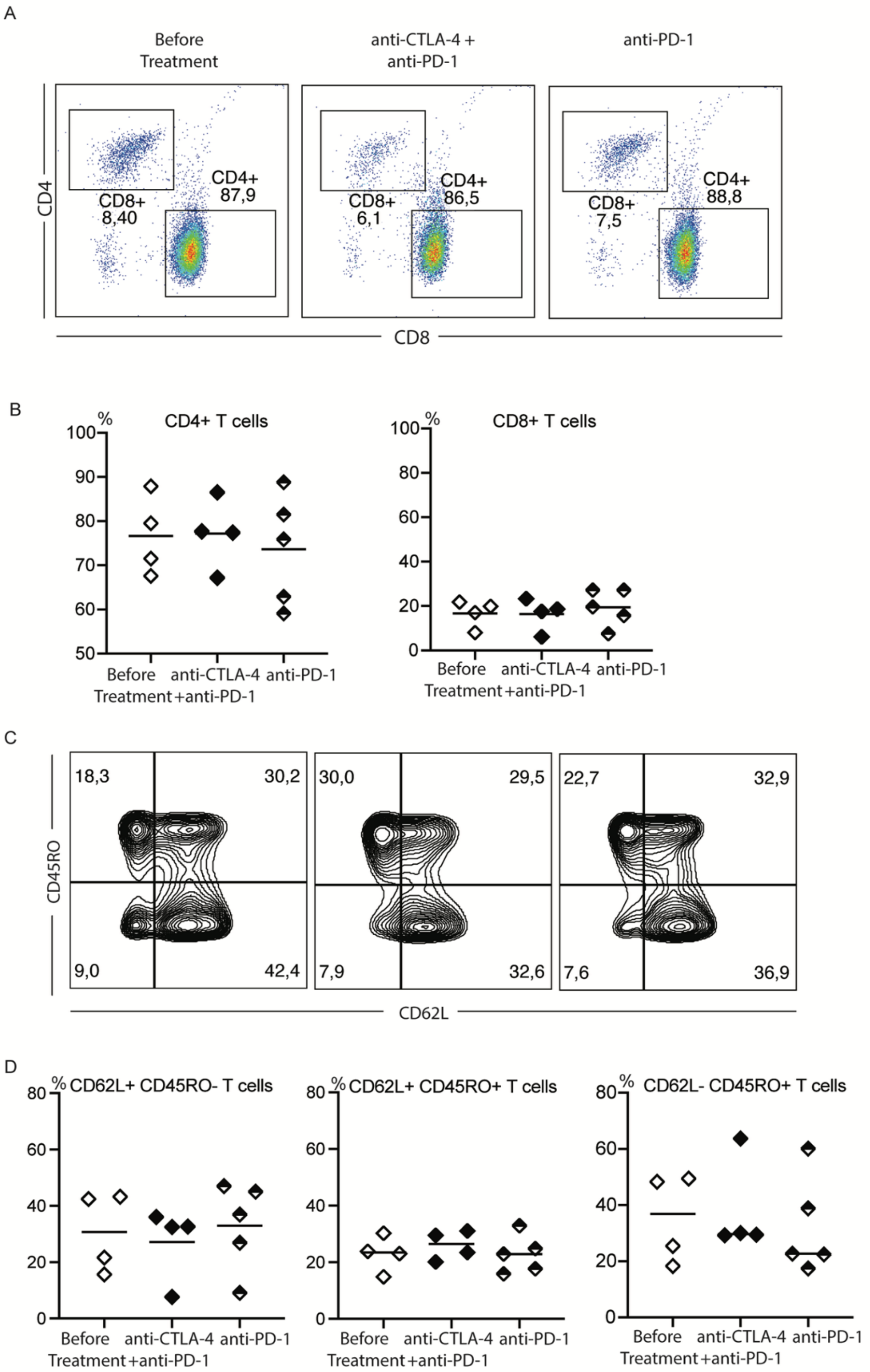

Supplemental Figure 7. Phenotype of circulating T cells in cancer patients treated with CPI. (**A**) Representative dot plots showing CD4 and CD8 expression on $CD3^+$ gated T cells at the indicated time points. (**B**) Frequencies of circulating $CD4^+$ and $CD8^+$ T cell populations are summarized as percentages. (**C**) Representative dot plots show CD45RO and CD62L surface expression on $CD4^+$ T cells at each time point and the frequency of each subset is represented as percentages in (**D**). Averages are shown with a bar.

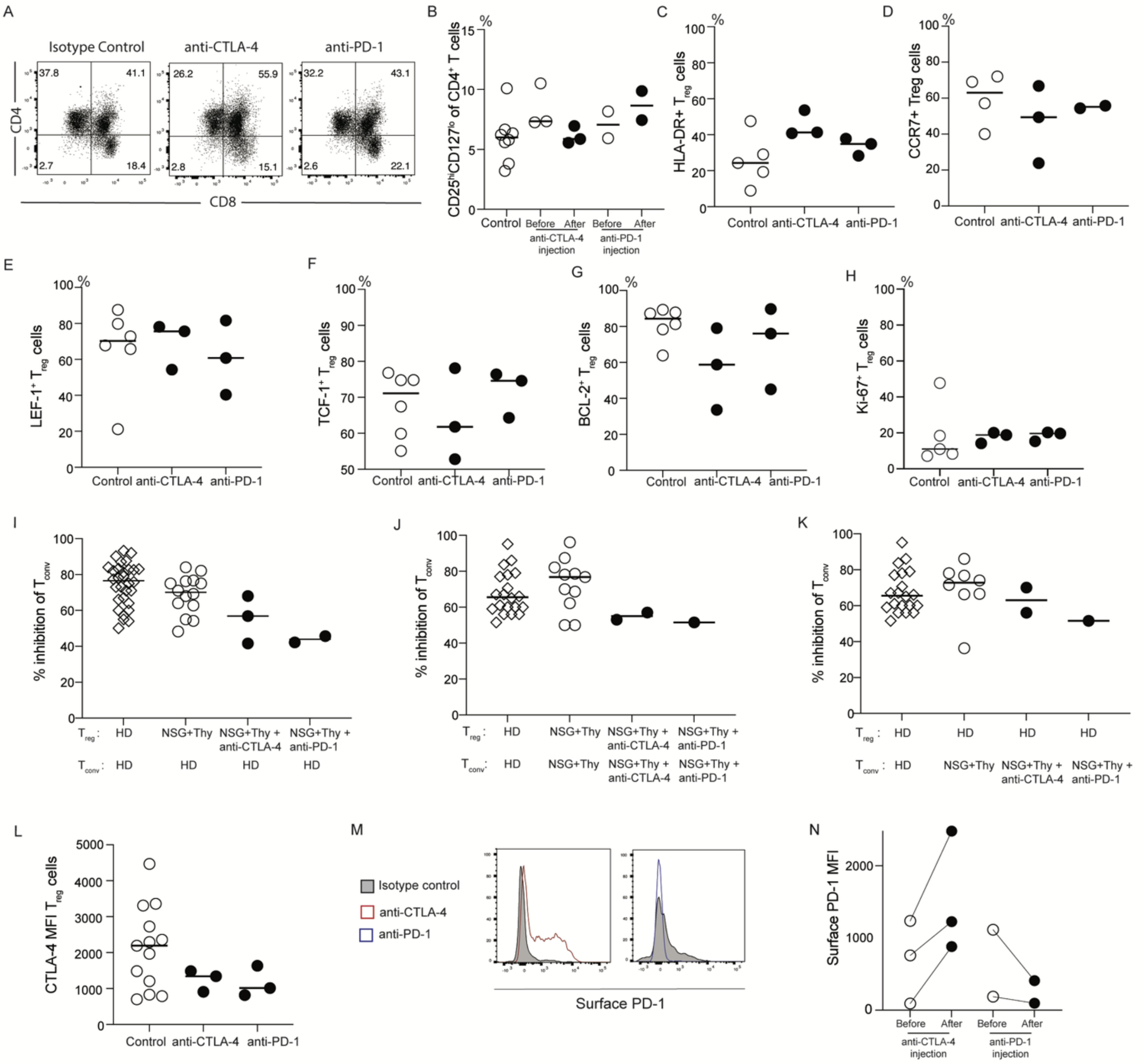

Supplemental Figure 8. Impact of anti-CTLA-4 and anti-PD-1 injections on T and B cell phenotype in humanized mice. (**A**) Representative dot plots show CD4 and CD8 expression on thymocytes isolated from the human autologous thymic graft co-transplanted in humanized mice injected with isotype control, anti-CTLA-4 or anti-PD-1. The frequencies of circulating $CD25^{hi}CD127^{lo}$ Treg in $CD3^{+}CD4^{+}$ T cells is represented in (**B**), and the proportion of HLA-DR^{+} (**C**), $CCR7^{+}$ (**D**), LEF-1^{+} (**E**), TCF-1^{+} (F), BCL-2^{+} (**G**) and Ki-67^{+} (**H**) splenic Tregs in the indicated panels. (**I-K**) Summaries of the suppressive capacity of Tregs from healthy donors (HDs) and NSG + thymus humanized mice injected with isotype control, anti-CTLA-4 or anti-PD-1, in autologous and heterologous settings. Averages are shown as bars. The effect of CPI injections on surface CTLA-4 expression on gated Treg cells (**L**), and surface PD-1 expression on gated Treg cells represented as histograms (**M**) and summarized as surface PD-1 MFI (**N**).

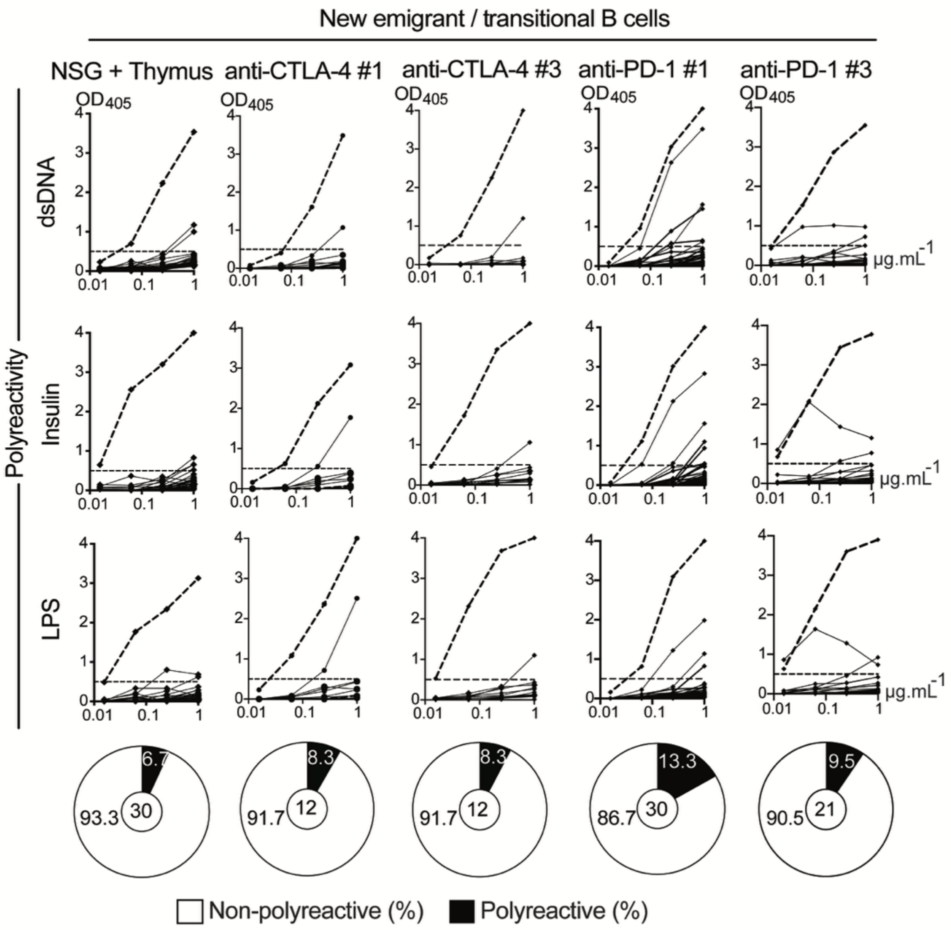

Supplemental Figure 9. Central B cell tolerance remains functional after anti-CTLA-4 and anti-PD-1 injections. Recombinant Abs cloned from single mature naïve B cells isolated from the indicated humanized mice were tested by ELISA for polyreactivity tested using dsDNA, insulin and LPS antigens. Dotted lines show the ED38 positive control. Horizontal lines show the cutoff OD_{405} for positive reactivity. For each humanized mouse, frequencies of non-reactive (white area) and reactive (black area) clones are summarized in a pie chart, with the total number of clones tested indicated in the centers.

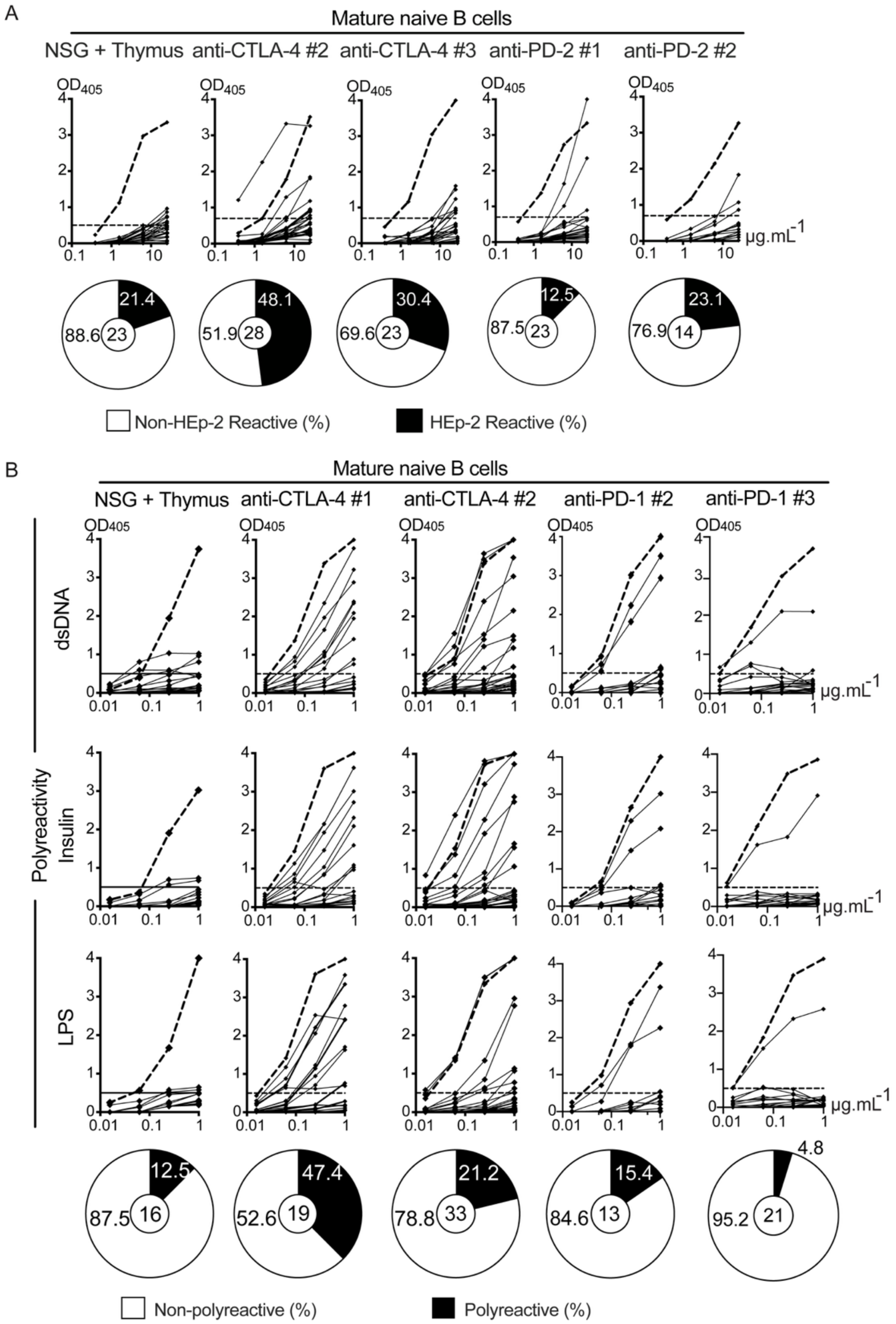

Supplemental Figure 10. Anti-CTLA-4 but not anti-PD-1 leads to the production of autoreactive mature naïve B cells. Recombinant Abs cloned from single mature naïve B cells isolated from the indicated humanized mice were tested by ELISA for anti–HEp-2 cell reactivity (**A**) and polyreactivity tested using dsDNA, insulin and LPS antigens (**B**). Dotted lines show the ED38 positive control. Horizontal lines show the cutoff OD_{405} for positive reactivity. For each humanized mouse, frequencies of non-reactive (white area) and reactive (black area) clones are summarized in a pie chart, with the total number of clones tested indicated in the centers.

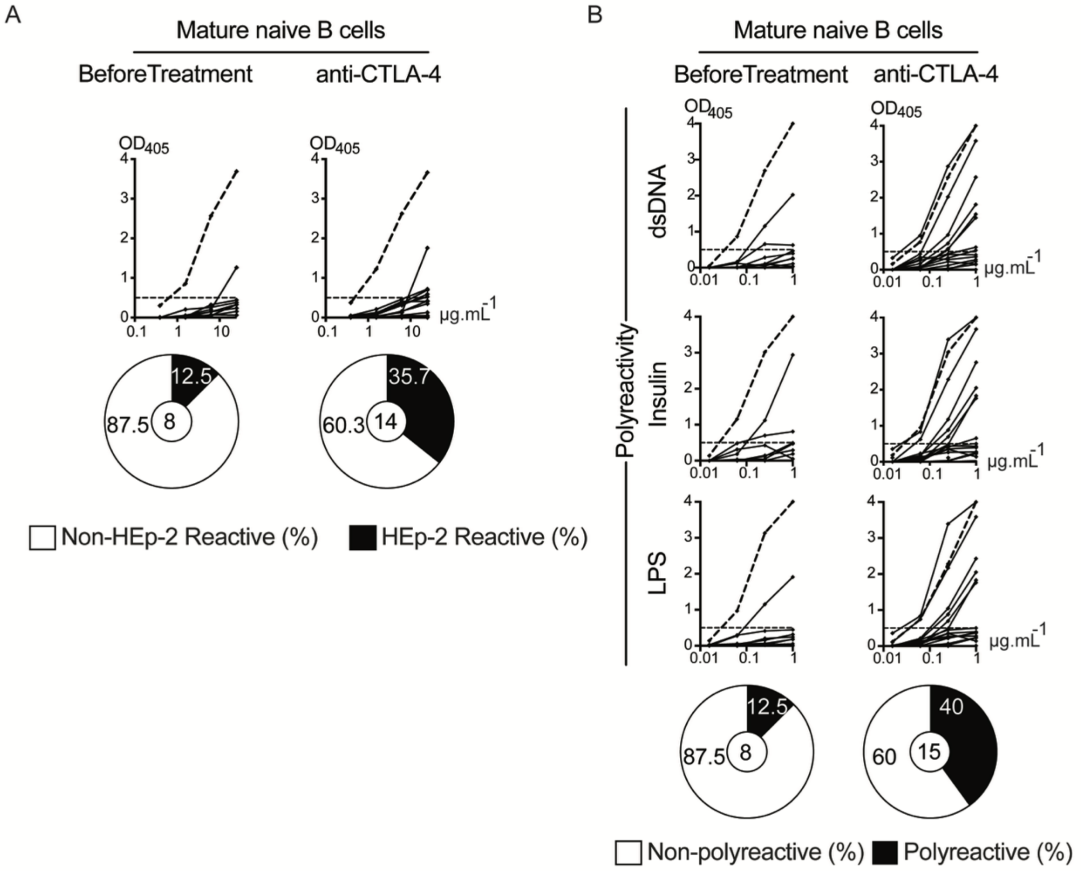

Supplemental Figure 11. Anti-CTLA-4 treatment alone is sufficient to induce the production of autoreactive mature naïve B cells in a cancer patient. Recombinant Abs cloned from single mature naïve B cells isolated from a cancer patient before and after anti-CTLA-4 monotherapy were tested by ELISA for anti–HEp-2 cell reactivity (**A**) and polyreactivity tested using dsDNA, insulin and LPS antigens (**B**). Dotted lines show the ED38 positive control. Horizontal lines show the cutoff OD_{405} for positive reactivity. The frequencies of nonreactive (white area) and reactive (black area) clones are summarized in pie charts, with the total number of clones tested indicated in the centers.

Supplemental Table 1. Characteristics of melanoma patients treated with CPIs

Patient Code	Sex	Cancer Diagnosis	Sample	Age	irAEs	Other treatment	Medical History
**Patient 134**	M	Melanoma	Before treatment	74	-	-	SSS, HTN, HLD, CAD s/p PCI, GERD, esophageal dysmotility, benign temporal mass resected
			Anti-CTLA-4 +anti-PD-1 treatment	74	Thyroiditis, hypothyroidism, vitiligo, mild diarrhea, uveitis	-	
			Anti-PD-1 treatment	75	Myalgias	-	
**Patient 145**	F	Melanoma	Before treatment	68	-	Left craniotomy and parietal mass resection	BCC, pseudogout, ADHD, Lyme disease, HTN, HLD
			Anti-CTLA-4 +anti-PD-1 treatment	69	Hypopituitarism dry eyes and dry mouth	-	
			Anti-PD-1 treatment	69	-	-	
**Patient 326**	M	Melanoma	Before treatment	39	-	-	-
			Anti-CTLA-4 +anti-PD-1 treatment	39	-	-	
			Anti-PD-1 treatment	39	Vitiligo, arthralgias, diarrhea, chills, myalgias, night sweats	-	
**Patient 196**	M	Melanoma	Before treatment	48	-	-	-
			Anti-CTLA-4 +anti-PD-1 treatment	48	Hypopituitarism	Solumedrol, maintenance prednisone	
			Anti-PD-1 treatment	49	Joint pain, pancreatic inflammation and elevated lipase	Short burst of steroids and solumedrol	
**Patient 081**	M	Melanoma	Anti-PD-1 treatment	75	Arthritis, AKI	Tonsillectomy	HTN, BCC, Squamous cell carcinoma, Actinic keratosis, HLD, BPH, Hiatal hernia
**Patient 066**	M	Melanoma	Anti-PD-1 treatment	66	Arthritis, diarrhea	-	OA

AKI: Acute kidney injury; SSS: Sick sinus syndrome; HTN: Hypertension; HLD: Hyperlipidemia; CAD: Coronary artery disease; PCI: Percutaneous coronary intervention; GERD: Gastroesophageal reflux disease; BCC: Basal cell carcinoma; ADHD: Attention-deficit/hyperactivity disorder; BPH: Benign prostatic hyperplasia; OA: Osteoarthritis.

Supplemental Table 2. Repertoire and reactivity of recombinant antibodies cloned from single new emigrant/transitional and mature naïve B cells from cancer patients and humanized mice.
See XL file.

Supplemental Table 3. List of human fetal samples used in the study.

**Fetus #**	**Age (days)**	**Sex**
FT25583	103	F
FT26172	137	F
FT27665	130	M
FT28514	113	M
FT27662	115	F
FT28534	117	M

Supplemental Table 4. Antibody reagents, dye and dilutions used in the study.

Supplier	Catalog No	Antigen	Fluor. or Conj.	Clone	Working Dilution
BD Biosciences	559867	CD21	APC	B-LY4	1:25
BD Cell Analysis	561381	CD21	V450	B-LY4	1:25
BioLegend	305205	CD80	FITC	2D10	1:25
BioLegend	311906	CD267(TACI)	PE	1A1	1:25
BioLegend	302612	CD25	PE-Cy7	BC96	1:10
BioLegend	302606	CD25	PE	BC96	1:25
BioLegend	312214	CD10	PE-Cy7	HI10A	1:10
BioLegend	310910	CD69	PE	FN50	1:25
BioLegend	310922	CD69	AF700	FN50	1:25
BioLegend	305412	CD86	APC	IT2.2	1:50
BioLegend	356408	CD27	PerCp-Cy5.5	M-T271	1:25
BD Biosciences	555782	IgM	FITC	MHM-88	1:10
BioLegend	302224	CD19	Pacific Blue	HIB19	1:50
BioLegend	302218	CD19	APC-Cy7	HIB19	1:10
BioLegend	302212	CD19	APC	HIB19	1:50
BD Cell Analysis	555787	Ig-G	PE	G18-145	1:50
BioLegend	329918	PD1	PE-Cy7	EH12.2H7	1:10
BioLegend	329920	PD-1	BV421	EH12.2H7	1:10
BioLegend	317418	CD4	APC-Cy7	OKT4	1:25
BioLegend	351318	CD127	APC	A019D5	1:25
eBioscience	83003742	CD3	e605NC	OKT3	1:50
BioLegend	304216	CD45RO	Pacific Blue	UCHL1	1:10
BioLegend	356910	CXCR5	PerCP-Cy5.5	J252D4	1:10
BioLegend	304803	CD62L	FITC	DRAK56	1:25
BD Cell analysis	555729	CD28	PE	CD28.2	1:10
BioLegend	300920	CD8	AF700	HIT8a	1:50
BioLegend	313510	ICOS	APC	C39A.4A	1:25
BioLegend	359118	CCR5	BV421	J418F1	1:25
BioLegend	351322	CD127	PerCP-Cy5.5	A019D5	1:25
R&D System	FAB197F-100	CCR7	FITC	150503	1:25
BioLegend	353741	CXCR3	AF700	G025H7	1:10
BioLegend	307626	HLA-DR	AF700	I.243	1:50
BioLegend	658709	BCL2	BV421	100	1:10
eBioscience	53-4776-41	FOXP3	FITC	PCH101	1:10
BioLegend	350504	Ki-67	PE	Ki-67	1:10
BioLegend	137218	HELIOS	AF647	22F6	1:10
Cell Signaling	14440S	LEF1	PE		1:25
Cell Signaling	6709S	TCF1	APC	C63D9	1:10
BD Cell Analysis	560939	CTLA4	PE	BNI3	1:10
BioLegend	329707	PD-L1	APC	29E283	1:10
BioLegend	329806	ICOS-L	PE	9F.8A4	1:25
